# Bumps on the Road: The Way to Clean Relaxation Dispersion Magic-Angle Spinning NMR

**DOI:** 10.1021/jacs.5c09057

**Published:** 2025-08-01

**Authors:** Ben P. Tatman, Vidhyalakshmi Sridharan, Motilal Uttarkabat, Christopher P. Jaroniec, Matthias Ernst, Petra Rovó, Paul Schanda

**Affiliations:** † 148492 Institute of Science and Technology Austria, Am Campus 1, 3400 Klosterneuburg, Austria; ‡ Department of Chemistry and Biochemistry, 2647 The Ohio State University, 100 West 18th Avenue, Columbus, Ohio 43210, United States; § Institute of Molecular Physical Science, 27219 ETH Zurich, 8093 Zurich, Switzerland

## Abstract

Microsecond-to-millisecond motions are instrumental for many biomolecular functions, including enzymatic activity and ligand binding. Bloch-McConnell Relaxation Dispersion (BMRD) Nuclear Magnetic Resonance (NMR) spectroscopy is a key technique for studying these dynamic processes. While BMRD experiments are routinely used to probe protein motions in solution, the experiment is more demanding in the solid state, where dipolar couplings complicate the spin dynamics. It is believed that high deuteration levels are required and sufficient to obtain accurate and quantitative data. Here we show that even under fast magic-angle spinning and high levels of deuteration artifactual “bumps” in ^15^N *R*_1ρ_ BMRD profiles are common. The origin of these artifacts is identified as a second-order three-spin Mixed Rotational and Rotary Resonance (MIRROR) recoupling condition. These artifacts are found to be a significant confounding factor for the accurate quantification of microsecond protein dynamics using BMRD in the solid state. We show that the application of low-power continuous wave (CW) decoupling simultaneously with the ^15^N spin-lock leads to the suppression of these conditions and enables quantitative measurements of microsecond exchange in the solid state. Remarkably, the application of decoupling allows the measurement of accurate BMRD even in fully protonated proteins at 100 kHz MAS, thus extending the scope of μs dynamics measurements in MAS NMR.

## Graphical abstract

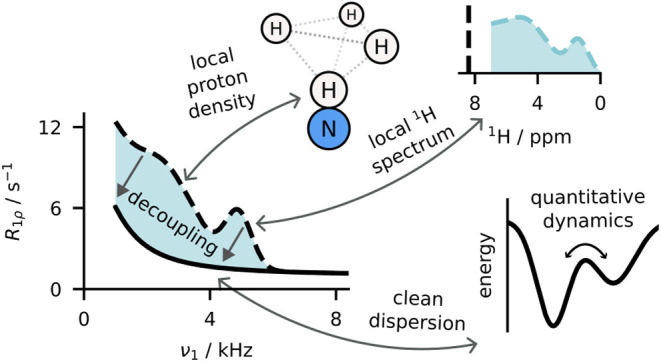

## Introduction

Motions occurring on a time scale of microseconds to milliseconds (μs-ms) play an important role in the function of numerous biological systems. Enzymatic turnover,^1^ transitions underlying allosteric communication^2^ and folding/unfolding of proteins often occur on these time scales. To understand the mechanisms of these functional processes, one ideally wants to characterize the involved states structurally and to quantify the kinetics of their exchange. While obtaining insights into these time scales by molecular dynamics (MD) simulations remains a significant challenge,^3,4^ a number of experimental techniques, such as Nuclear Magnetic Resonance (NMR), optical, infrared or terahertz spectroscopies, electron microscopy and crystallography, can probe such motions.^5–7^ NMR stands out among these methods because it can probe motions that occur at equilibrium without perturbing the system, and it can do so with a resolution of individual nuclear spins.^8,9^ The success of NMR in characterizing dynamics at high accuracy is largely rooted in a sophisticated arsenal of methods available today. These methods aim to quantitatively probe dynamics while avoiding artifactual contributions, such as evolution of nuclear spin states due to effects other than dynamics. The development of optimized pulse sequences in combination with a careful choice of the targeted atomic sites, possibly combined with suitable isotope labeling, has been instrumental to achieve this goal.^10,11^

One class of methods that have proven particularly powerful for probing μs-ms motions are the Bloch-McConnell Relaxation Dispersion (BMRD) type experiments.^10,11^ In an *R*_1ρ_ BMRD experiment, the rotating-frame transverse relaxation rate constant of a nuclear spin (*R*_1ρ_) is measured as a function of an applied spin-lock RF field (ν_1_ = *γB*_1_). When the RF field is weak, fluctuations of the spin’s isotropic chemical shift on μs-ms time scales result in elevated *R*_1ρ_ relaxation rate constants. This contribution to the relaxation is increasingly quenched toward greater spin-lock fields. By fitting a suitable model to the experimentally determined relaxation-dispersion curve, it is possible to interrogate the rates at which the involved processes occur, and, in some cases and under certain experimental conditions, extract populations and chemical-shift differences between exchanging sites.^12,13^ The range of spin-lock field strengths over which such effects are observed depends on the chemical-shift difference of the involved states and the kinetics of the motion; for ^15^N nuclei, BMRD effects are commonly observed when the RF field strength ν_1_ is below several kHz.

In magic-angle spinning (MAS) solid-state NMR, coherent contributions to the spin evolution, in particular due to dipolar couplings to ^1^H spins, complicate quantitative dynamics measurements because they alter the apparent *R*_1ρ_ rate constants. Depending on the MAS frequency and type of isotope labeling, the apparent decay may be dominated by such dipolar dephasing, thus masking the dynamics contribution, a situation that has long hampered studies of protein dynamics by *R*_1ρ_ BMRD measurements. Extensive deuteration and fast MAS are believed to largely suppress these effects, based on the observation of (near-)­flat *R*_1ρ_ RD profiles for many residues. Building upon this observation, quantitative *R*_1ρ_ BMRD analyses of dynamics have been shown by us^14–21^ and others.^22–27^

Several recent ^15^N *R*_1ρ_ BMRD MAS NMR studies have, however, observed the presence of unexpected “bump”-like features in the measured dispersion profiles. These effects have remained unexplained and have not been further considered.^15,24^ Such features may also be found unnoticed in several other publications,^14,17–23,25–27^ noting that these are often difficult to detect owing to the limited number of data points typically recorded. Several representative examples of such features, both from published work and from new data generated in this study, are shown in Figure 1, all obtained on deuterated proteins and 40–60 kHz MAS. We note that these artifacts appear over a range of samples prepared by different research groups and measured with different instruments and experimental conditions (*B*_0_ field strengths and MAS frequencies). They are, therefore, unlikely to have arisen due to issues in the experimental setup or errors in sample preparation or equipment malfunction. As is clear from Figure 1, these features lead to significant distortions of the dispersion curves, and consequently preclude the possibility of quantitative data analysis (*vide infra*). As will be shown in the following, in protonated samples these effects become massive, and, possibly for this reason, quantitative BMRD studies of protonated proteins have not yet been reported.

**Figure 1:**
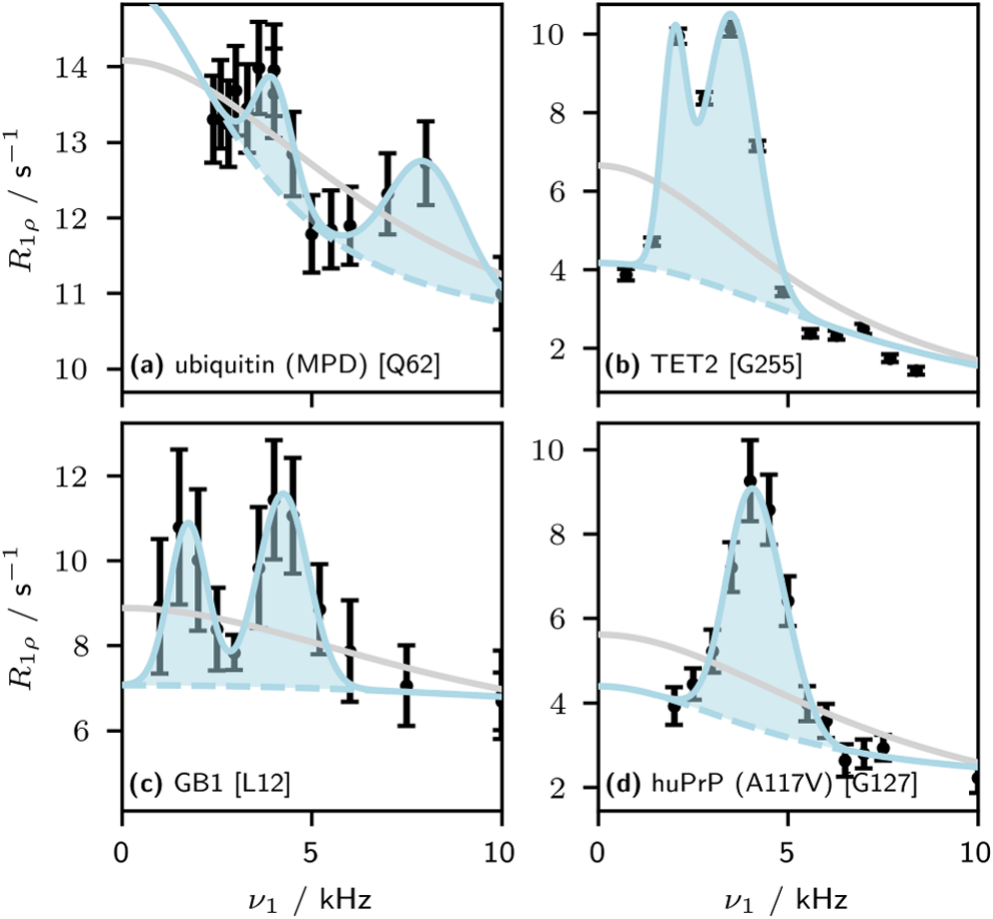
Several examples of artifacts observed in solid-state NMR ^15^N Bloch-McConnell relaxation dispersion curves. The residue number is given in square brackets. Gray lines show fits of a two-site exchange model to the data, while blue lines show a two-site exchange model with the addition of a phenomenological model of the artifacts as a visual guide. In all cases, the protein samples were perdeuterated and back-exchanged with ^1^H_2_O. (a) ubiquitin crystallized in MPD.^15^ (b) TET2 (this work). (c) Microcrystalline GB1 with 2 mM Gd­(DTPA-BMA).^24^ (d) huPrP23-144 A117 V mutant (this work).^28^

In this paper, we systematically investigate the origin of these “bump” features. Through experiments performed over a range of magnetic field strengths (600 and 700 MHz ^1^H Larmor frequency), magic-angle spinning (MAS) frequencies (55.56 and 100.0 kHz), and protonation levels (protonated and perdeuterated/back-exchanged samples), we identify the bump features as arising from a second-order Mixed Rotary and Rotational Resonance (MIRROR) condition,^29,30^ whereby irradiation of the nuclear spins of interest at the chemical shift difference of two adjacent protons leads to a recoupling of the heteronuclear dipolar coupling. The recoupling-induced dephasing leads to an apparent increase in the relaxation rate. We demonstrate that this condition is responsible for the generation of *pseudo*-dispersion profiles leading to an overestimation of the number of sites undergoing microsecond motions. Moreover, it is the major cause of the large coherent contribution in fully protonated samples, which makes BMRD analysis of such samples essentially impossible. We find that the application of proton decoupling simultaneously with the spin lock on the spin of interest (analogously to that previously applied at slower spinning^31,32^ ) disrupts this polarization-transfer process and thus enables the measurement of accurate relaxation dispersion profiles, thus allowing the quantification of microsecond motion, even in fully protonated protein samples at ≈100 kHz MAS. Moreover, we also address another previous bottleneck: the experimental time required to measure a BMRD experiment. We show that employing one-point measurements of BMRD experiments, similar to those commonly used in solution-state NMR, leads to largely accelerated experiments, which allows for measurements of the dispersion profiles at a large number of RF field strengths.

## Results

### Investigating the Origin of Bump Artifacts with Accelerated One-Point BMRD Measurements

Previously employed MAS NMR BMRD measurements involved collecting a time series of 2D spectra at each RF field strength, whereby the spin-lock duration was incremented and the time dependency of intensities fitted to an exponential decay.^14–26^ Due to the long experimental time required, the number of RF field strengths with this scheme is typically limited to ≤10. We sought to adopt a scheme similar to that often used in solution-state NMR RD measurements,^33^ whereby only one single relaxation delay is measured for each RF field strength. Unlike in solution, though, relaxation in solids is always multiexponential because crystallites oriented differently experience different relaxation decay,^34,35^ and we investigated whether this would affect the applicability of the one-point measurement. As discussed in the Supporting Information (SI) (Section 2), the one-point approach to BMRD allows quantitative measurements of BMRD profiles. To obtain absolute values of *R*_1ρ_ from the one-point series, we additionally measured a full spin-lock duration series for a few selected RF field strengths (see SI, Section 1).

Equipped with a time-efficient way to measure *R*_1ρ_, we collected BMRD profiles for ^15^N ubiquitin crystals that were either deuterated or protonated (both in 100% H_2_O based buffer) at 64 RF field strengths (unless otherwise specified—see the SI for full details). Because of the speed of the one-point method, such a measurement requires only ca. 24–38 h. Figure 2a,b show that the frequency at which the bumps occur is proportional to the applied external static magnetic field strength. This implies that the cause is likely related to chemical shifts or differences thereof. We additionally note that the bumps do not appear at the same spin-lock frequency for each residue and that several bumps at different amplitudes may appear for a single site. Figure 2c,d show that the bumps appear at both 55.56 and 100.0 kHz MAS frequencies, and there does not appear to be a direct relationship between MAS frequency and bump intensity. However, it is interesting to note a shift in the frequency of the bump with changing MAS frequency. This suggests that the artifact may relate to the presence of an anisotropic interaction which is impacted by MAS, yet not completely removed. Given that a spinning rate of 100 kHz is in excess of any anisotropic interaction present which would be able to influence the apparent relaxation (the highest likely being the vicinal CH_2_^1^H–^1^H dipolar coupling on the order of 22 kHz), this points to the presence in the spin Hamiltonian of a homogeneous higher-order term, likely involving protons.

**Figure 2:**
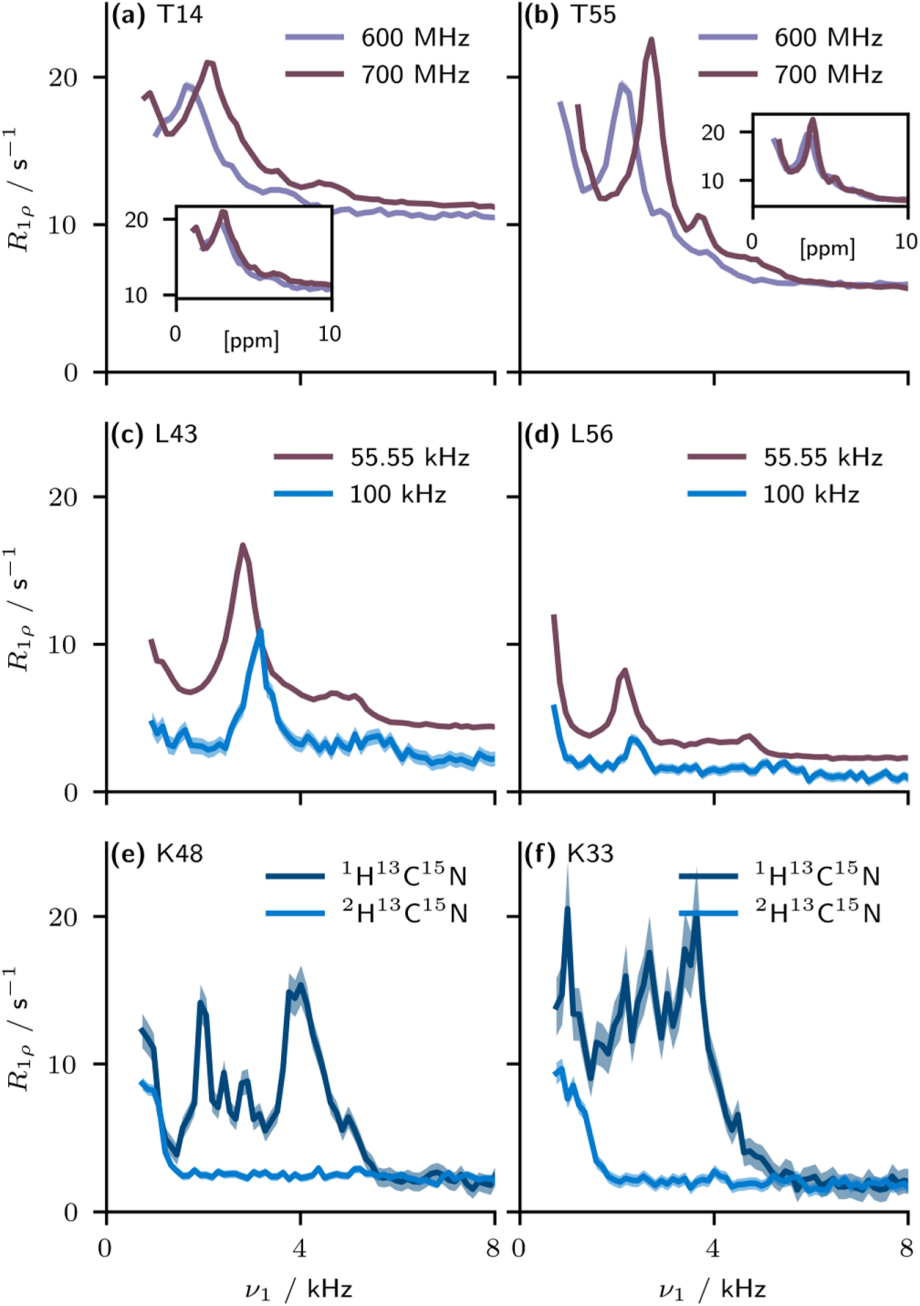
Variation of the observed artifacts under different experimental conditions. (a, b) Measurements made at 55.56 kHz MAS on perdeuterated ubiquitin (with ^13^C_δ_/^2^H_*βγ*_/^15^N_ϵ_ labeled arginine^36^) at two magnetic fields (note that owing to probe head limitations, the 600 MHz measurements were made at a higher temperature than those at 700 MHz). Inset axes show the same data, only with the frequency axis in units of ^1^H ppm (that is, divided by the ^1^H Larmor frequency). (c, d) Dispersion curves at 700 MHz (^1^H Larmor frequency) on perdeuterated ubiquitin at 55.56 and 100.0 kHz MAS. (e, f) Dispersion curves at 100.0 kHz MAS, 700 MHz, on protonated and perdeuterated ubiquitin (both with ^13^C_δ_/^2^H_*βγ*_/^15^N_ϵ_ labeled arginine^36^). Uncertainty bars are illustrated at ±1 standard deviation. The SI contains a full set of dispersion curves for each condition given here.

The necessity of protons for the artifact to be observed is further confirmed by the results shown in Figure 2e,f, where dispersion curves are compared for fully protonated and perdeuterated ubiquitin at 100 kHz MAS. Namely, in the protonated sample, elevated rate constants are observed over a considerable range of frequencies. Interestingly, these extend only to a certain frequency, beyond which they precipitously drop off; the frequency at which this dropoff occurs is residue dependent, and the “plateau” of *R*_1ρ_ at RF field strengths exceeding approximately 6 kHz is similar in both samples.

One such recoupling condition which matches these observed lines of evidence is the Mixed Rotational and Rotary Resonance condition (MIRROR).^29,30^ This recoupling condition arises in a three-spin *I*_2_ *S* system when the *S* spin is irradiated with a radio frequency field with an amplitude equal to the isotropic chemical shift difference of the *I* spins:

1 $$\nu_{1}^{S}=n\nu_{r}\pm \left|\nu_{0}^{\mathrm{IA}}-\nu_{0}^{\mathrm{IB}}\right|$$

where ν_0_ is the resonance frequency of the given spin, ν_1_ the applied rf field amplitude, ν_*r*_ the MAS frequency, and |*n*| ≤ 4. The specific spins are indicated by the superscripts. Matching this condition leads to a recoupled effective second-order Hamiltonian of the form:

2 $$Ĥ=\omega_{\mathrm{eff}}\left({Î}_{+}^{A}{Î}_{-}^{B}{Ŝ}_{-}+{Î}_{-}^{A}{Î}_{+}^{B}{Ŝ}_{+}\right)$$

where *Î*_+_, *Î*_–_, *Ŝ*_+_, and *Ŝ*_–_ represent the raising and lowering operators for spins *I* and *S*, respectively. Expressions for the effective frequency can be found in Scholz et al. (2008)^29^ (eq 12). The effect of this Hamiltonian is shown in Figure 3a; irradiation of the *S* spin (in this case, ^15^N) at the isotropic chemical shift difference of the *I* spins (here, ^1^H) matches the *n* = 0 MIRROR condition and leads to differential polarization of the *I* spins, and an apparent decay of magnetization on the *S* spin.

**Figure 3:**
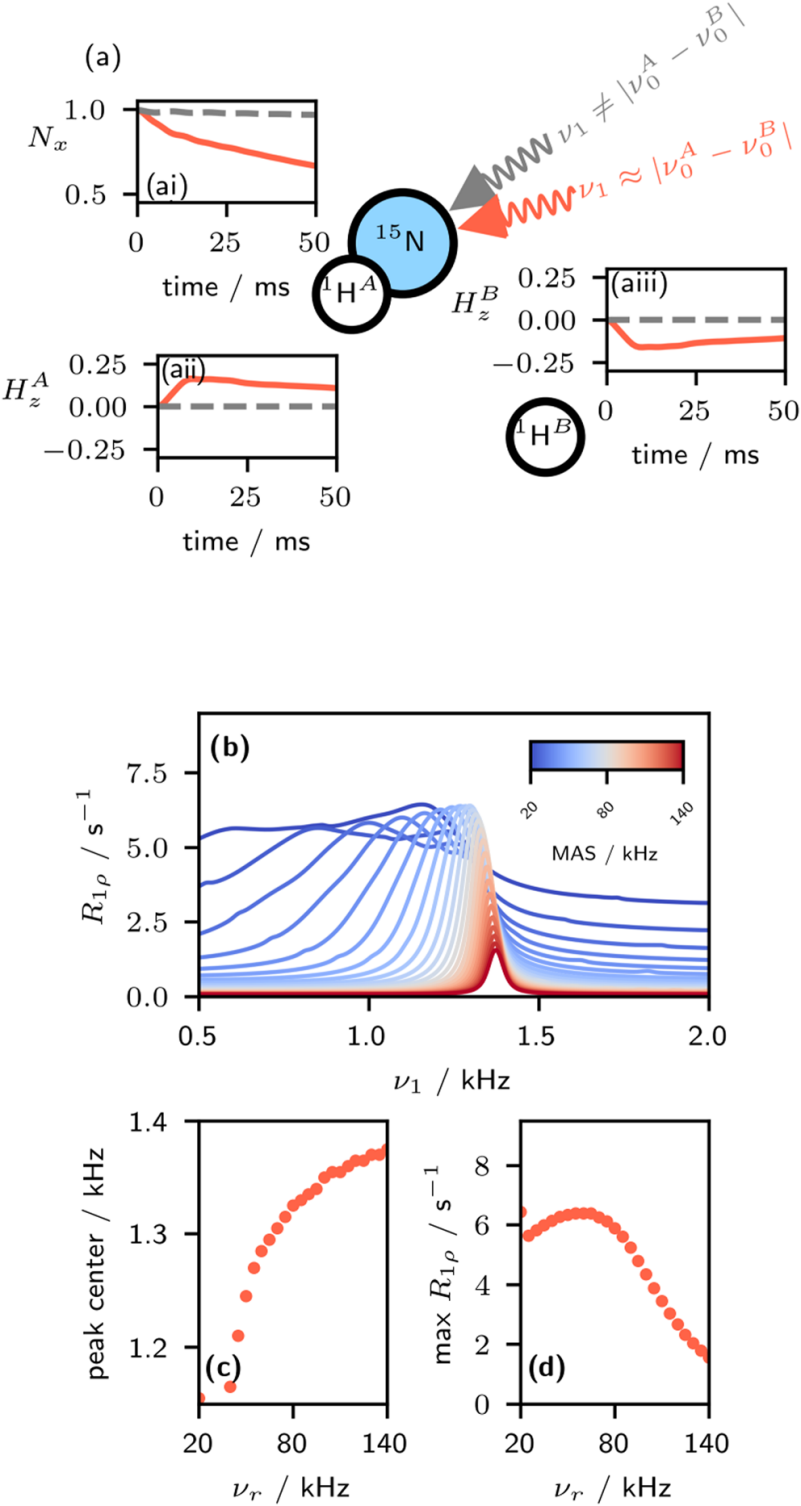
Simulations of the bump artifact. (a) Illustrative spin system. The chemical shifts of the ^1^H spins were set at −700 and +700 Hz, and 30 s^–1^ of random field relaxation was applied to them to represent the effect of the bulk proton spin bath (note that in the absence of this random field relaxation, there is insignificant contribution to the dispersion). Simulations are shown both under irradiation at the MIRROR condition (1270 Hz (shifted from 1400 Hz owing to the MAS dependence), solid red) and not at the MIRROR condition (100 Hz, dashed gray). These simulations were performed at 55.56 kHz MAS. (ai) evolution of the transverse ^15^N magnetization. (aii,aiii) evolution of the longitudinal magnetization on the two protons. (b) effective *R*_1ρ_ measured under irradiation of the ^15^N at frequency ν_1_. (c) Frequency at which the bump artifact occurs as a function of spinning frequency, ν_*r*_. (d) Maximum *R*_1ρ_ rate constant as a function of spinning frequency.

### Simulations and Experiments Support MIRROR as the Origin of the Bump Artifact

Using spin dynamics simulations, we investigated whether the decay in *S* spin magnetization occurring at the MIRROR recoupling conditions could explain the experimentally observed increase in ^15^N *R*_1ρ_ (Figure 3). In these simulations, we observed the MIRROR recoupling condition as an increase in *R*_1ρ_ at an RF field closely matching the chemical shift difference of the ^1^H spins. Interestingly, the specific frequency at which the elevated *R*_1ρ_ rates (the “bump”) occurs, appears to shift to lower frequencies (Figure 3c) at lower MAS rates, and is asymptotic toward the chemical-shift difference of the two ^1^H spins as the MAS rate increases (a value of 1.4 kHz was set in the simulation for this chemical-shift difference). The MAS-dependent frequency shift qualitatively matches the trend we observed experimentally (see Figure 2c,d). This MAS dependency is most likely caused by second-order fictitious fields generated by the dipolar coupling under MAS and an applied spin lock that have isotropic and anisotropic contributions and increase with decreasing MAS frequency.^37^ These features may be analogous to similar features in the HORROR peak frequency observed by Krushelnitsky et al. (2023).^32^ The simulations also suggest a decrease in the artifactual *R*_1ρ_ with increasing MAS rate (Figure 3d). We do not see as large of a decrease experimentally (see, e.g., Figure 2c). We suspect that this arises due to the *in-silico* treatment of the proton spin bath; in simulations, we enforce a constant 30 s^–1^ random-field relaxation on the ^1^H spins. Experimentally, however, the reduction in ^1^H–^1^H spin diffusion at faster MAS^38^ would likely lead to MAS-dependent ^1^H relaxation, with some sites experiencing faster or slower relaxation. This may offer an explanation for the observed inconsistency.

We also investigated an experimental case in which we can identify which specific protons are involved in the MIRROR condition. In the perdeuterated ubiquitin samples used here, we would expect that the only protons present in significant quantities in the sample are those which undergo exchange with water, i.e., backbone amides, and side chain −OH and −NH groups. The only exchangeable protons that have chemical shifts in the region of 4–5.5 ppm, and are expected to have a sufficiently long residence time for dipolar transfer, are the OH protons of the serine and threonine residues.^39^ In one region of ubiquitin, visualized in Figure 4, there exist four such groups: S20, S57, T22, T55. Investigating the relaxation dispersion profiles recorded for four nearby amide sites, we observe “bump”-like artifacts. While the spin-lock frequency at which these artifacts occur varies depending on the specific site, we find that significant artifacts are located at a frequency commensurate with the chemical shift difference between the respective amide ^1^H and a ^1^H at a chemical shift of ≈5 ppm, typical for serine or threonine hydroxyl ^1^H.^39^ That these artifacts appear for sites in close proximity to such residues supports the conclusion that the artifacts arise as a consequence of the MIRROR recoupling conditions.

**Figure 4:**
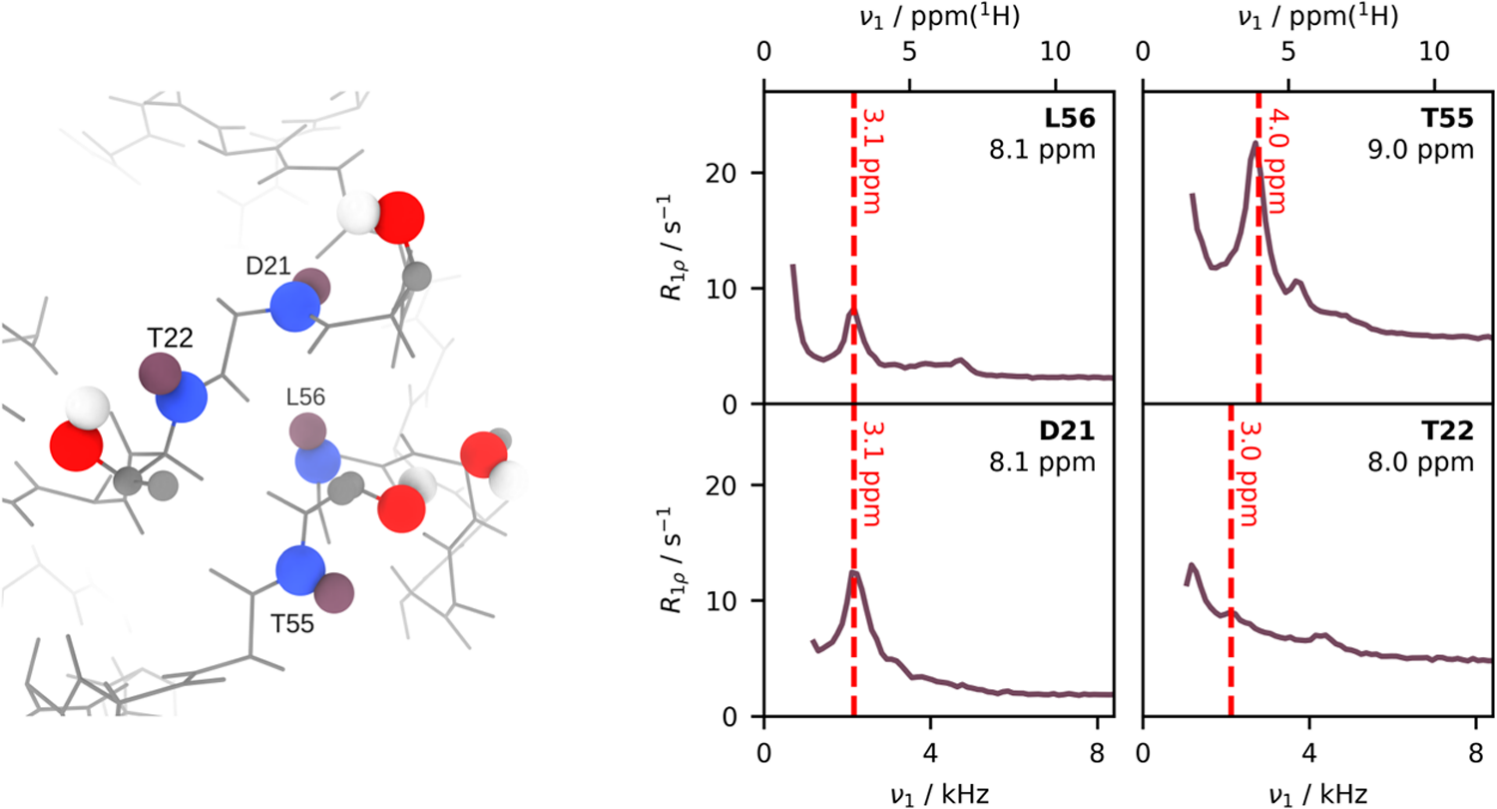
Dispersion profiles for sites in close proximity to serines and threonines. Left: The local structure surrounding S20, T22, T55, S57 (PDB: 3ONS^40^). Right: Selected dispersion profiles (measured at a ^1^H Larmor frequency of 700 MHz and under 55.56 kHz MAS) in close proximity to this region. The difference in chemical shift between the adjacent amide ^1^H and 5 ppm, a frequency typical of Thr/Ser OH groups, is indicated by a dashed red line. Uncertainty bars are illustrated at ±1 standard deviation.

While we have only considered “bumps” arising from the MIRROR condition with ^1^H nuclei, it is of course possible that these could also arise with other heteronuclei such as ^13^C and ^2^H. We suspect, however, that these will be less prevalent; in the case of ^13^C, the slower relaxation relative to ^1^H would lead to a reduction in the bump amplitude, while for ^2^H the low chemical shift dispersion, combined with the lower gyromagnetic ratio, would lead to these bumps forming at lower spin-lock frequencies.

### Suppressing the Artifactual Bumps

Based on the likely cause of the bump artifacts, we can now address methods to suppress them. For the measurement of *R*_1ρ_ under slower MAS conditions, high-power decoupling ^1^H is commonly applied to remove the influence of the ^1^H spins.^31^ However, under the fast MAS conditions used here, high-power CW decoupling is not feasible due to other detrimental first-order recoupling conditions. It would require the application of at least 200 kHz ^1^H RF field strength to avoid these recoupling conditions. Because the required relaxation delays are typically tens to hundreds of millseconds, the decoupling would likely lead to significant probe and sample damage. Low-power ^1^H decoupling during the ^15^N spin lock, on the other hand, runs the risk of introducing other recoupling conditions or enabling cross-relaxation pathways.^32^ Figure 5a shows simulations in the absence of decoupling, with 8 kHz CW decoupling, and with 16 kHz CW decoupling. Both 8 and 16 kHz CW decoupling remove the bump artifacts in simulations. However, in the 8 kHz case, a second-order cross-polarization match condition is apparent in the simulation at a spin lock amplitude of ≈8 kHz,^41^ rendering the reliable measurement of the ^15^N *R*_1ρ_ rate constant impossible. We recommend a minimum ν_1_^*H*^ of at least 1.5× the maximum applied ν_1_^*N*^, while additionally avoiding the ν_1_^*H*^ + ν_1_^*N*^ = ν_*r*_ condition (this condition may also lead to a significant cross-relaxation contribution). It is also recommended to avoid the HORROR condition at ν_*r*_/2.

**Figure 5:**
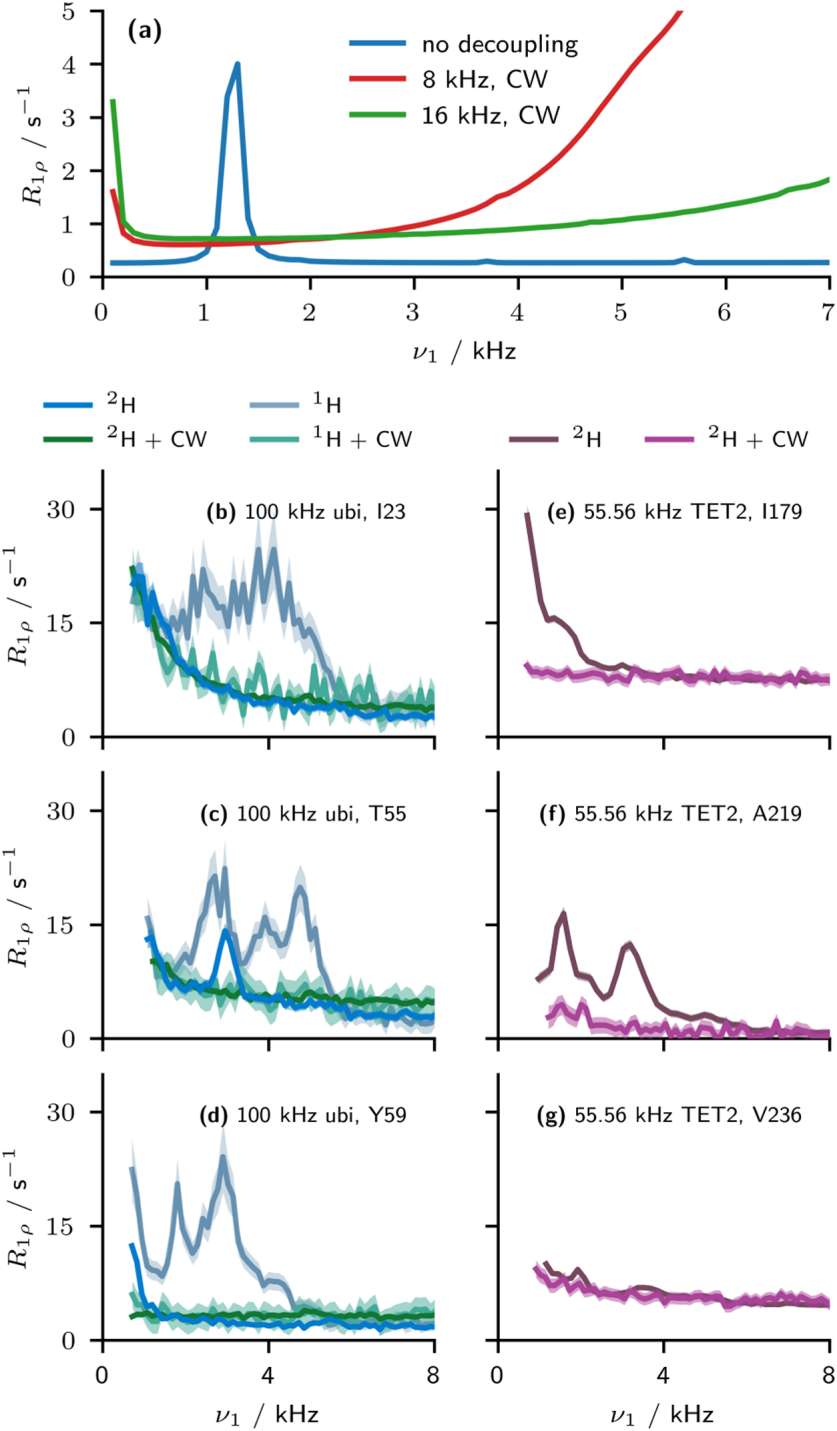
Application of decoupling to remove the bump artifact. (a) GAMMA simulations of the effect of low power CW decoupling on the bump artifact. (b–d) Comparison of dispersion curves measured in protonated and perdeuterated ubiquitin, with or without 16 kHz CW ^1^H decoupling at 100.0 kHz MAS. (e–g) Comparison of dispersion curves measured in perdeuterated TET2 at 55.56 kHz with and without 16 kHz CW decoupling (note that only 48 frequencies were recorded for nondecoupled TET2). Uncertainty bars are illustrated at ±1 standard deviation.

In addition to continuous-wave ^1^H decoupling, we explored the use of a composite pulse decoupling scheme, two-pulse phase-modulated (TPPM) decoupling.^42^ Additional systematic recoupling conditions were found experimentally with these schemes, which we attribute to the periodic nature of the decoupling sequence and additional interferences (see the SI, Section 3). With this in mind, we chose to proceed with a 16 kHz CW decoupling condition at both 55.56 and 100.0 kHz MAS, where no resonance conditions are to be expected for the range of ^15^N spin-lock field strengths required for collecting the BMRD profiles.

Figure 5b–g shows the application of 16 kHz CW decoupling during the measurement of relaxation dispersion in both perdeuterated and protonated ubiquitin at 100.0 kHz MAS (Figure 5b–d), and perdeuterated TET2 at 55.56 kHz MAS (Figure 5e–g). While the application of decoupling will necessarily change the spectral density sampling for the anisotropic contribution to *R*_1ρ_,^32^ and thus may affect the baseline between the experiments, *R*_1ρ,0_, the decoupling appears to completely remove the artifactual bumps under both conditions. We find that the apparent dispersion with decoupling, both in the protonated and perdeuterated samples, is less than or equal to the dispersion contribution to the curve in the nondecoupled perdeuterated case. In Figure 5b,d,e,g, we show examples where the nondecoupled dispersion curves for the perdeuterated system do not show any obvious bump artifacts. For Figure 5b,g, the absence of a bump artifact is confirmed as the application of CW decoupling does not produce significant differences compared to the nondecoupled dispersion curves in the perdeuterated samples. However, in the examples shown in Figures 5d and e, the application of decoupling leads to a significant change, indicating that the apparent dispersion was rather a bump artifact and not a consequence of microsecond time scale motions. In this case, it is likely that the apparent dispersion actually arises due to MIRROR conditions between nearby amide protons, where the difference in chemical shifts would be less than or similar to the lowest applied nutation frequency. That it is not possible to distinguish these two possibilities without the application of decoupling indicates that dispersion curves measured in the absence of decoupling, as has typically been performed to date, can lead to a significant misinterpretation as to the nature of the dynamics occurring within the system.

We additionally performed experiments on fully protonated ubiquitin at 100.0 kHz with and without CW decoupling (Figure 5b–d). We find that even in this more challenging case, the decoupling is able to remove the artifacts. The resulting dispersion curves in protonated ubiquitin under decoupling show no significant variation from those measured in perdeuterated ubiquitin for spin-lock fields ≥1.5 kHz, indicating that ^15^N BMRD is suitable for protonated samples at high MAS frequencies (≥100.0 kHz) when CW ^1^H decoupling is also applied during the ^15^N spin lock.

### The Excess *R*_1ρ_ Reports on the Local Proton Environment

The artifactual contribution of the MIRROR condition to the measured *R*_1ρ_ arises due to recoupling to ^1^H spins in spatial proximity. Consequently, the difference of the BMRD profile with and without decoupling could be useful as a probe of this local chemical environment. By taking the difference of the decoupled and nondecoupled relaxation dispersion curves,

3 $$\Delta_{1\rho }\left(\nu_{1}\right)=R_{1\rho }^{\mathrm{non}‐\mathrm{decoupled}}\left(\nu_{1}\right)-R_{1\rho }^{\mathrm{decoupled}}\left(\nu_{1}\right)$$

we obtain a frequency-dependent measurand Δ_1ρ_ (Figure 6a). In principle, owing to the different sampling of the spectral densities in the anisotropic contribution to *R*_1ρ,0_, this metric may include an additional offset, which may explain why in some cases (e.g., Figure 6e) the resulting Δ_1ρ_ plateaus at a negative value. Assuming that one of the two ^1^Hs involved in the MIRROR recoupling condition is the directly bonded amide proton, we can relate the RF frequency to an effective chemical shift of the other proton involved in the interaction. Using a structural model and previously assigned side chain and backbone chemical shifts of ubiquitin (from solution state),^43–45^ we fit a phenomenological model to Δ_1ρ_ profiles measured in protonated ubiquitin at 100.0 kHz MAS (see eq 13 in the Supporting Information). In this, we fit all residues simultaneously with four parameters, relating to the amplitude, peak width (noting that this will additionally be influenced by RF field inhomogeneity across the sample), distance scaling, and MAS-dependent frequency scaling (as identified in simulation, Figure 3c, likely arising due to the presence of higher-order fictitious fields). Despite the small number of fit parameters, and a number of ^1^H sites without assignments, we find remarkably good agreement between this model and the experimental profiles (Figure 6b–e, also see SI). We additionally applied the model directly to the perdeuterated ubiquitin without further optimization, and found that while the agreement is less accurate, it is still able to match many of the artifactual low-frequency components. The model indicates a distance scaling, 1/*r* ^*a*^ of *a* = 3.72 ± 0.01, which suggests that at an exact match condition, a ^1^H spin ≈3.5 Å away would give a Δ_1ρ_ contribution of 1 s^–1^. Although we are not proposing to use this as a method for determining structure, it demonstrates that far from being a nuisance, the artifactual bumps may provide site-specific structural and spectral insight into the local proton environment around a spin.

**Figure 6:**
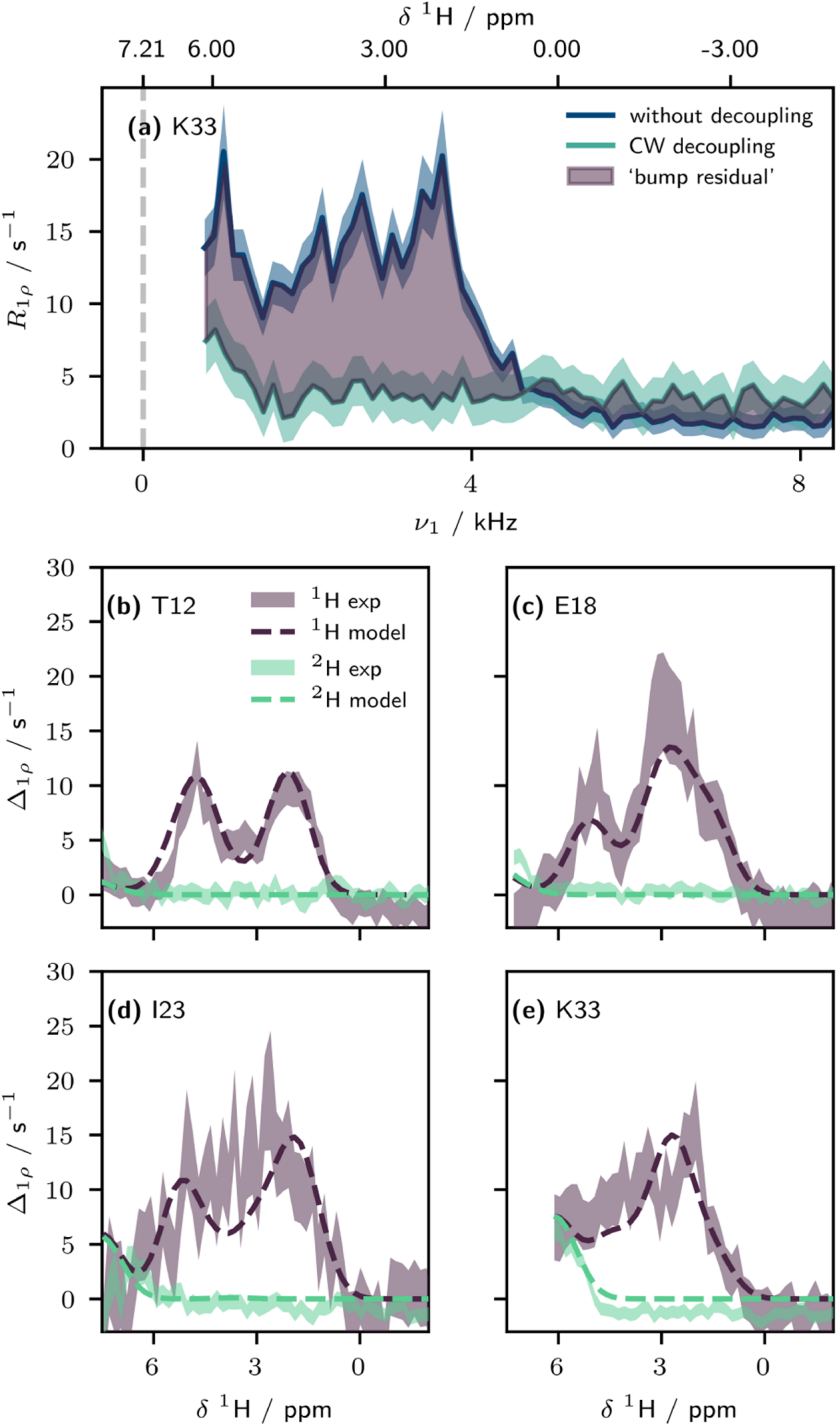
Modeling of the artifactual bump residual, Δ_1ρ_, according to the local chemical environment of the nuclear spin. (a) Assuming that one of the protons involved in the three-spin MIRROR recoupling is the directly bound amide proton, the spin-lock RF frequency can be related to a ^1^H chemical shift of the other spin involved. Subtracting the decoupled BMRD profile from the nondecoupled profile (in this case, for ^1^H ubiquitin at 100.0 kHz MAS) gives a “bump residual”. (b–d) Comparison of experimental “bump residuals” (^1^H (purple) and ^2^H (green) ubiquitin (with arginine labeling, see Methods) at 100.0 kHz MAS) with a four parameter model based on a structural model of ubiquitin (PDB: 3ONS). Uncertainty bars are illustrated at ±1 standard deviation.

### Importance of Removing the Artifacts for Quantification of Kinetics

Relaxation dispersion profiles measured in the presence of the bump artifacts can lead to significant misinterpretation of exchange processes, as these do not reflect the true dispersion. To identify possible implications this has for previous studies which have used BMRD to study the dynamics of proteins, we fit here a two-site exchange model to dispersion curves measured (ν_1_ > 1.7 kHz) in both protonated and perdeuterated ubiquitin at 100.0 kHz MAS, both with and without CW decoupling. Specifically, we fit the equation:

4 $$R_{1\rho }\left(\nu_{1}\right)=\frac{\phi_{\mathrm{ex}}k_{\mathrm{ex}}}{k_{\mathrm{ex}}^{2}+{\left(2\pi \nu_{1}\right)}^{2}}+R_{1\rho ,0}$$

where we fit *k*_ex_ as a global parameter (noting that this must be divided by 2π to convert it into s^–1^) and the scaling factor ϕ_ex_ and baseline *R*_1ρ,0_ as site-specific parameters. While we make use of a two-site exchange model here, this should not be interpreted as evidence that the exchange in this case is a two-site process; the use of this model here is purely for evaluation purposes, given the relative ubiquity of this model in solid-state analyses of exchange.^10,14,22,24^ Figure 7 shows how the quantification of microsecond exchange in ubiquitin at 100.0 kHz MAS is impacted by the artifacts. Both the quantification of exchange rate constant (Figure 7d) and site-specific exchange amplitude ϕ_ex_ (Figure 7e) are impacted by the presence of the bump. Application of CW decoupling, both to protonated and perdeuterated ubiquitin, enables the site-specific quantitation of exchange processes; in the absence of decoupling, the resulting kinetic parameters may be significantly misleading. For example, residue 51 (Figure 7b) appears to undergo exchange in both protonated and perdeuterated ubiquitin. Under application of CW decoupling, however, it becomes apparent that the dispersion profile is flat within experimental uncertainty. Consequently, analyses of exchange processes made on nondecoupled BMRD profiles, whether in protonated or perdeuterated samples, may be misleading and are unsuitable for interpretation. It should be noted that the dynamic process previously reported for perdeuterated ubiquitin is still reasonable: residues 23, 27, and 55 as reported before have nonflat BMRD profiles. The exchange rate constants determined previously,^19^ *k*_ex_ of 1800 s^–1^ (reported there in units of s rad^–1^) are, however, close to values found here in decoupled experiments. This may be due to the quite large BMRD of residue 23, which makes the effect of the bumps less consequential. However, several sites which were previously reported to show significant dispersion may now be seen to have been artifactual: for example, Ma et al. (2014) found residues 51 and 52 to have significant dispersions which may now be seen to have arisen due to bump artifacts.^14^

**Figure 7:**
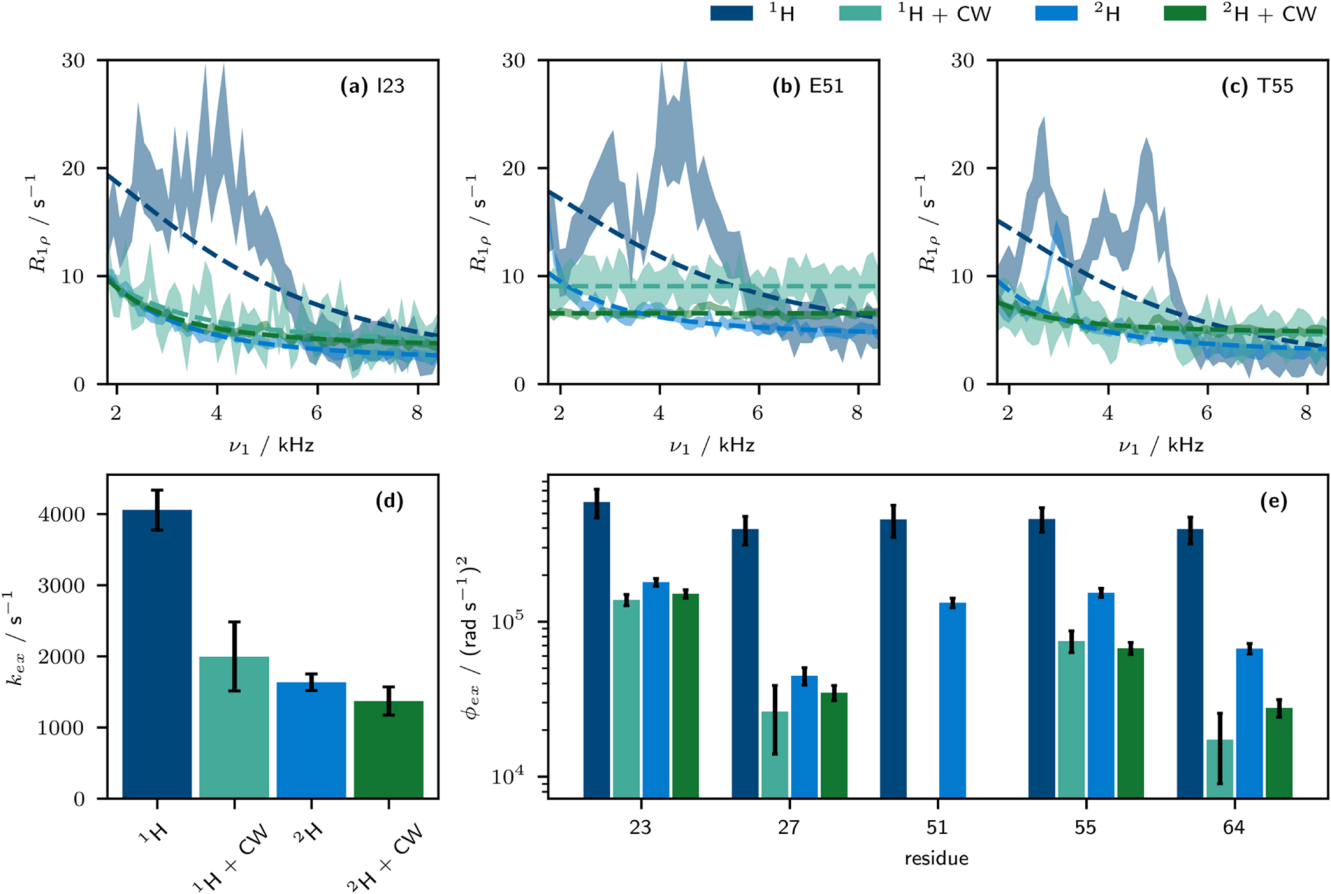
Consideration of the impact of not accounting for the bump artifact. Relaxation dispersion profiles measured at 100.0 kHz MAS in perdeuterated and protonated ubiquitin with and without decoupling were analyzed separately. For each case, all well-resolved sites were fit with a single *k*_ex_ value. A two-site exchange model was used for all sites in a two step fitting process; first, all residues were included. Then, any residues for which ϕ_ex_ < 10^5^ (rad s^–1^)^2^ had ϕ_ex_ set to 0. The results of the second fitting step are shown. (a–c) Relaxation dispersion profiles with fit models shown as dashed lines. (d) The resulting global fit *k*_ex_ values for each experimental setup. (e) The resulting ϕ_ex_ values for a selection of residues.

This analysis additionally highlights that even protonated samples are amenable to quantitatively accurate BMRD studies in the presence of ^1^H decoupling (at MAS frequencies of 100 kHz), which has not previously been possible.

## Conclusions

Artifactual bumps have been observed in solid-state ^15^N BMRD profiles for several proteins. We have systematically investigated the origin of these artifacts and identified them as arising due to the second-order MIRROR recoupling condition. This condition is found to be a significant confounding factor for the quantification of exchange processes using BMRD, to the extent that it precludes quantitative analysis of exchange and leads to the incorrect quantification of exchange-related parameters. To prevent these systematic artifacts, we recommend the application of CW decoupling on the ^1^H channel simultaneously with the spin lock on ^15^N. We additionally note that this three-spin recoupling condition may also affect NEar Rotary Resonance Dispersion (NERRD) experiments, as these may match the *n* = 1 condition in eq 1 (see Section 7 of the SI for more discussion of such effects).

## Experimental Section

### Sample Preparation

Ubiquitin was produced by bacterial overexpression with ^13^C, ^15^N isotope labeling (protonated sample) and ^2^H, ^15^N labeling. The samples additionally comprise specific ^13^C^1^H_2_ labeling of the δ carbon of Arg residues with the other carbons of the Arg side chain deuterated and ^13^C, as described by Rohden et al. (2025).^36^ This labeling was not of importance for the present study. Ubiquitin was crystallized with MPD as described elsewhere^19^ and packed into 0.7 and 1.3 mm rotors. The ^2^H, ^13^C, ^15^N labeled TET2 samples were produced and packed into 1.3 mm rotors as described by Napoli et al. (2024).^46^

### Solid-State NMR

MAS NMR experiments were performed using Bruker Avance Neo spectrometers operating at ^1^H Larmor frequencies of 600 and 700 MHz, using (at both fields) 1.3 mm probes and (at 700 MHz) a 0.7 mm probe tuned to ^1^H, ^13^C, ^15^N frequencies. For 1.3 mm experiments, the VT temperature was set to 233 and 245 K for experiments at 700 and 600 MHz, respectively. The internal sample temperature for the 700 MHz 1.3 mm experiments was calibrated to be 308 K. 0.7 mm experiments were performed at a VT temperature of 273 K, which was adjusted such that the internal sample temperature was approximately the same as in the 700 MHz 1.3 mm experiments, with the resonance frequency of the water signal appearing at the same shift in a ^1^H 1D spectrum. In all experiments, ^1^H–^15^N out-and-back cross-polarization (CP) experiments were used to record relaxation rates in a 2D ^15^N–^1^H manner using ^1^H detection. The pulse programs used were adapted from those in ssNMRlib.^47^ Specific details, including relaxation delays, pulse programs, and power levels, are detailed in Table 1 in the SI.

### Relaxation Analysis

The resulting NMR spectra were processed using TopSpin 4.1.4, with each having a Gaussian window function applied of 5 Hz in the direct and 2 Hz in the indirect dimensions. Peaks were picked and the intensities determined using nmrglue 0.10.^48^ Any peaks for which significant overlap was observed were omitted from the analysis to exclude the possibility that artifactual bumps arose from errors in deconvolution. For instance, if a fast-relaxing site is close in space to a slower relaxing site, the deconvolution algorithm may switch to the wrong site between different spectra of the series. While this may be restricted by enforcing stricter bounds on the peak position, this may add greater error to the fit. In each spectrum, sites for which the peak integration did not converge were omitted. Additionally, in the profiles we omitted measurements where there was a sudden jump of >7 s^–1^ in the resulting relaxation rate, as these relate to poor convergence. The noise was estimated using the standard deviation of a region of the spectra containing no peaks.

We determined relaxation rate constants in two ways. Traditionally in solid-state NMR, transverse spin-lock relaxation rate constants are determined by recording the decay of magnetization, *I*(*t*; ν_1_^′^), at a given applied spin-lock frequency, ν_1_^′^, as a function of time *t*. Typically, a monoexponential decay is fit to the decaying magnetization:

5 $$I\left(t;\nu_{1}^{\prime }\right)=I\left(0;\nu_{1}^{\prime }\right)\exp\left(-R_{1\rho }^{\prime }\left(\nu_{1}^{\prime }\right)t\right)$$

In our analysis here, we used the SciPy 1.14.1 curve_fit function to fit eq 5 to the experimentally determined decay curves, where the σ parameter was set to the noise level in the measurements such that the resulting covariance matrix was scaled accordingly to the experimental noise. The uncertainty in the resulting *R*_1ρ_^′^(ν_1_), *u*(*R*_1ρ_^′^(ν_1_)), was estimated using covariance matrix resulting from this fit.

It is necessary to correct both the applied nutation frequency ν_1_^′^ and fit *R*_1ρ_^′^ for the offset between the frequency of the transmitter, ν_rf_, and the ^15^N Larmor frequency of a given site, ν_0_^*N*^:^10^

6 $$R_{1\rho }\left(\nu_{1}\right)=\frac{R_{1\rho }^{\prime }\left(\nu_{1}\right)-R_{1}\;\cos^{2}\left(\theta \right)}{\sin^{2}\left(\theta \right)}$$

$$\nu_{1}=\sqrt{\nu_{1}^{\prime 2}+{\left(\nu_{\mathrm{rf}}-\nu_{0}^{N}\right)}^{2}}$$ 7

where $\theta =\arctan\left(\frac{\nu_{1}^{\prime }}{\nu_{\mathrm{rf}}-\nu_{0}^{N}}\right)$ .

In the analysis performed in this paper we were interested in the fine-scale behavior of the relaxation dispersion curves. To investigate this in detail requires the measurement of *R*_1ρ_(ν_1_) at a significant number of nutation frequencies, under many different conditions. It is infeasible to record a relaxation dispersion curve with sufficiently many points using the traditional method. Instead, we took inspiration from solution-state NMR RD methods in which typically only two points are recorded. Here, we record a ^15^N–^1^H spectrum after applying a constant length spin-lock pulse as the frequency of this spin-lock pulse is varied systematically. The length of this spin-lock pulse should be calibrated to give a good balance of signal-to-noise while still remaining in a region of linear decay. In the case of samples with a wide dispersion of relaxation rates, it may be beneficial to measure two separate spin-lock pulse lengths to adequately quantify both components.^49^ It is necessary to correct the measured intensities, *I*_meas_(ν_1_^′^; *t*), for the angle the effective spin-lock field makes with the initial magnetization. At the end of the CP from ^1^H onto the ^15^N, the magnetization lies in the transverse plane as the nutation frequency of the CP on ^15^N is significantly greater than the transmitter offset to a given peak. However, the applied spin-lock field may be of a similar order-of-magnitude to this transmitter offset. Consequently, any magnetization not parallel to this spin-lock field will average out. A similar consideration then needs to be accounted for when the magnetization aligned along this spin-lock is then projected back onto the transverse plane. As a result, the “offset corrected” intensity may be obtained as

8 $$I\left(\nu_{1}^{\prime };t\right)=\frac{I_{\mathrm{meas}}\left(\nu_{1}^{\prime };t\right)}{\sin^{2}\left(\theta \right)}$$

where θ is as defined above. In the SI we show a comparison between profiles where the alignment is accounted for in this manner to profiles where the magnetization is explicitly aligned along the spin lock axis, where we find there is no significant difference. The resulting scaled intensity is then related to an offset *R*_1ρ_^′^(ν_1_^′^) as

9 $$\left[R_{1\rho }^{\prime }\left(\nu_{1}^{\prime }\right)+\mathrm{offset}\right]=-\frac{1}{t}\ln\left(I\left(\nu_{1}^{\prime };t\right)\right)$$

10 $$u\left([R_{1\rho }^{\prime }\left(\nu_{1}^{\prime }\right)+\mathrm{offset}]\right)=\frac{1}{t}\frac{u\left(I\left(\nu_{1}^{\prime };t\right)\right)}{I\left(\nu_{1}^{\prime };t\right)}$$

where the exponential prefactor of the decay curve gives rise to the offset term. It is assumed in this that the uncertainty in the time, *u*(*t*), is negligible with respect to the uncertainty in the measured peak intensities. While in the solution-state it is possible to determine this offset factor by recording the intensity at a separate time-point, the inherent multiexponential nature of the decay of magnetization in the solid-state leads to significant uncertainties if the analysis is performed in this manner. Instead, we determine the offset through comparison with a sparser set of measurements made in the full manner with a full set of decay curves, as described above. This enables both a reduction in the uncertainty of this offset term, and enables us to validate that the deviation in the curves from monoexponentiality is not significant enough to disrupt the analysis (see the SI for a further discussion). Specifically, we interpolate the values of [*R*_1ρ_^′^(ν_1_^′^) + offset] to the frequencies ν_1_^′^ of *R*_1ρ_^′^(ν_1_) values determined in the method described above. The offset term is then determined as

11 $$\mathrm{offset}=\mathrm{median}\;\left(\left[R_{1\rho }^{\prime }\left(\nu_{1}^{\prime }\right)+\mathrm{offset}\right]-R_{1\rho }^{\prime }\left(\nu_{1}^{\prime }\right)\right)$$

The offset is then removed:

12 $$R_{1\rho }^{\prime }\left(\nu_{1}^{\prime }\right)=\left[R_{1\rho }^{\prime }\left(\nu_{1}^{\prime }\right)+\mathrm{offset}\right]-\mathrm{offset}$$

which is then corrected for transmitter frequency offset in the manner given in eq 6. For ubiquitin, the *R*_1_ used for the correction in this analysis was measured at 850 MHz and taken from Schanda et al. (2010).^50^ In TET2, the *R*_1_ used for the correction was measured at 600 MHz. In this analysis, data for which θ ≤ 60° was omitted. It is not possible to measure such decoupled *R*_1_ rates owing to the long relaxation decays required. In general, however, *R*_1_ ≪ *R*_1ρ_, and so for θ ≤ 30° this correction is expected to be minimal.

## Supplementary Material



## Data Availability

All data supporting the conclusions of this article will be made available on ISTA Research Explorer (https://www.doi.org/10.15479/AT-ISTA-19696).

## Notes

One-point measurements, specific experimental details, pulse sequences, additional modeling, and a full set of dispersion curves (PDF)
