# Supplementary material for: Bumps on the Road: The Way to Clean Relaxation Dispersion Magic-Angle Spinning NMR
Source: J Am Chem Soc. 2025 Aug 1;147(32):29315–26. doi: 10.1021/jacs.5c09057 (PMC12356580; doi:10.1021/jacs.5c09057)
Supplement: Supplementary file 1 [file ja5c09057_si_001.pdf]

Supporting Information

# Bumps on the Road: The Way to Clean Relaxation Dispersion Magic-Angle Spinning NMR


Ben P. Tatman*[a], Vidhyalakshmi Sridharan[b], Motilal Uttarkabat[b], Christopher P. Jaroniec[b], Matthias Ernst[c], Petra Rovó[a] and Paul Schanda*[a]

[a] Ben P. Tatman*, P. Rovó, P. Schanda*
Institute of Science and Technology Austria, Am Campus 1, 3400 Klosterneuburg, Austria
E-mail: benjamin.tatman@ista.ac.at, paul.schanda@ista.ac.at

[b] Vidhyalakshmi Sridharan, M. Uttarkabat, C. P. Jaroniec
Department of Chemistry and Biochemistry, The Ohio State University, 100 West 18th Avenue, Columbus, Ohio 43210, USA

[c] Matthias Ernst
Institute of Molecular Physical Science, ETH Zurich, 8093 Zurich, Switzerland


## Contents


**1 Experimental Conditions** **2**

**2 Validity of Single Point Relaxation Measurement in the Solid State** **7**

**3 Alternate Decoupling Schemes** **10**

**4 Modelling the Bumps by Consideration of the Local Chemical Environment** **10**

**5 Using Alignment Pulses Before/After Spin Lock** **12**

**6 Representative GAMMA Simulation Input Files** **12**

**7 Potential for bump-like artefacts in NERRD profiles** **15**

**8 All Dispersion Curves** **17**

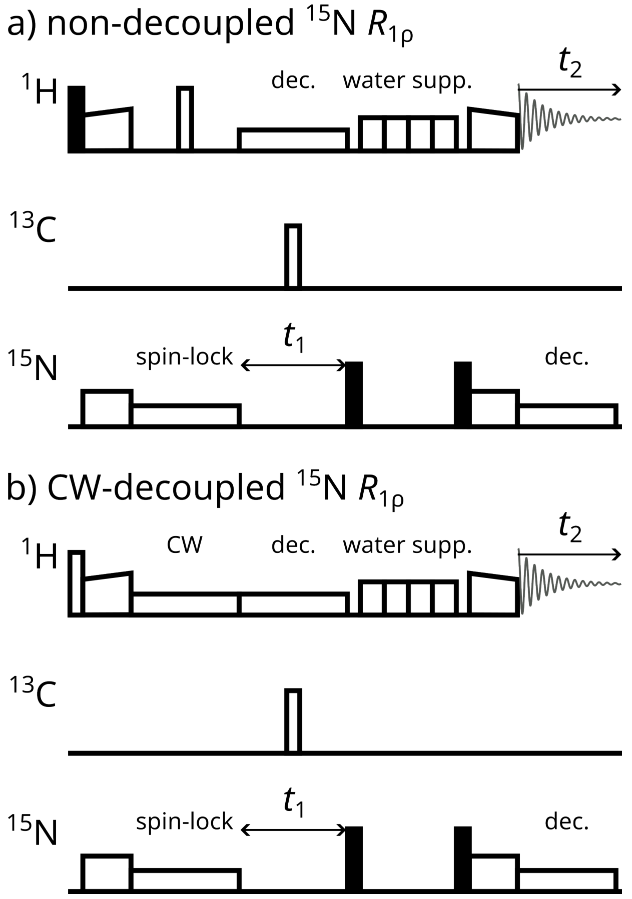


**Figure S1:** Pulse program. 90° pulses are shown as narrow, filled rectangles, while 180° pulses are shown as narrow, empty rectangles. Cross-polarisation pulses are shown with ramps on $^1$H.

# 1 Experimental Conditions

| | |
|---|---|
| $^2$H TET2 | **cross polarisation** |
| 700 MHz | *out:* $^1$H: 74.9 kHz (OPTO shape with $\beta = 63$, $\gamma = -125$), $^{15}$N: 17.5 kHz, 1.0 ms. |
| 55.5 kHz | *back:* $^1$H: 74.9 kHz (OPTO shape), $^{15}$N: 17.5 kHz, 1.0 ms. |
| non-decoupled | **composite pulses** |
| | *water suppression CPD:* 120.0 ms @ 10.0 kHz |
| | *$^1$H decoupling*: waltz16, 25.0 µs @ 10.0 kHz |
| | *$^{15}$N decoupling*: waltz16, 50.0 µs @ 5.0 kHz |
| | **dimensions** |
| | *$^1$H:* 56 scans, 29.8 ppm |
| | *$^{15}$N:* 112 points, 33.0 ppm |
| | **spin lock** |
| | **fast mode:** |
| | *frequencies (kHz):* 0.7, 0.9, 1.0, 1.2, 1.4, 1.5, 1.7, 1.8, 2.0, 2.2, 2.3, 2.5, 2.7, 2.8, 3.0, 3.2, 3.3, 3.5, 3.6, 3.8, 4.0, 4.1, 4.3, 4.5, 4.6, 4.8, 5.0, 5.1, 5.3, 5.5, 5.6, 5.8, 5.9, 6.1, 6.3, 6.4, 6.6, 6.8, 6.9, 7.1, 7.3, 7.4, 7.6, 7.7, 7.9, 8.1, 8.2, 8.4 |
| | *pulse length:* 100.0 ms |
| | **full decays:** |
| | *frequencies (kHz):* 0.7, 1.4, 2.1, 2.8, 3.5, 4.2, 4.9, 5.6, 6.3, 7.0, 7.7, 8.4 |
| | *spin-lock durations (ms):* 2.0, 5.0, 10.0, 25.0, 50.0, 100.0 |

| | |
|---|---|
| $^{2}$H TET2<br>700 MHz<br>55.5 kHz<br>16 kHz CW-decoupled | **cross polarisation**<br>*out:* $^{1}$H: 87.0 kHz (ramp90100.100), $^{15}$N: 40.0 kHz, 1.0 ms.<br>*back:* $^{1}$H: 87.0 kHz (ramp10090.100), $^{15}$N: 40.0 kHz, 1.0 ms.<br>**composite pulses**<br>*water suppression CPD:* 20.0 ms @ 16.0 kHz<br>*$^{1}$H decoupling*: swftppm, 50.0 µs @ 10.0 kHz<br>*$^{15}$N decoupling*: swftppm, 100.0 µs @ 5.0 kHz<br>**dimensions**<br>*$^{1}$H:* 32 scans, 29.8 ppm<br>*$^{15}$N:* 112 points, 33.0 ppm<br>**spin lock**<br>**fast mode:**<br>*frequencies (kHz):* 0.7, 0.8, 0.9, 1.1, 1.2, 1.3, 1.4, 1.6, 1.7, 1.8, 1.9, 2.0, 2.2, 2.3, 2.4, 2.5, 2.7, 2.8, 2.9, 3.0, 3.1, 3.3, 3.4, 3.5, 3.6, 3.8, 3.9, 4.0, 4.1, 4.2, 4.4, 4.5, 4.6, 4.7, 4.9, 5.0, 5.1, 5.2, 5.3, 5.5, 5.6, 5.7, 5.8, 6.0, 6.1, 6.2, 6.3, 6.4, 6.6, 6.7, 6.8, 6.9, 7.1, 7.2, 7.3, 7.4, 7.5, 7.7, 7.8, 7.9, 8.0, 8.2, 8.3, 8.4<br>*pulse length:* 40.0 ms<br>**full decays:**<br>*frequencies (kHz):* 1.9, 4.2, 6.5, 8.9, 3.1<br>*spin-lock durations (ms):* 1.0, 10.0, 20.0, 40.0, 60.0, 80.0, 110.0, 150.0 |
| $^{2}$H ubiquitin<br>700 MHz<br>55.5 kHz<br>non-decoupled | **cross polarisation**<br>*out:* $^{1}$H: 91.0 kHz (ramp90100.100), $^{15}$N: 38.0 kHz, 1.0 ms.<br>*back:* $^{1}$H: 91.0 kHz (ramp90100.100), $^{15}$N: 38.0 kHz, 1.0 ms.<br>**composite pulses**<br>*water suppression CPD:* 160.0 ms @ 16.0 kHz<br>*$^{1}$H decoupling*: waltz16, 25.0 µs @ 10.0 kHz<br>*$^{15}$N decoupling*: waltz16, 50.0 µs @ 5.0 kHz<br>**dimensions**<br>*$^{1}$H:* 16 scans, 29.8 ppm<br>*$^{15}$N:* 128 points, 33.0 ppm<br>**spin lock**<br>**fast mode:**<br>*frequencies (kHz):* 0.7, 0.8, 0.9, 1.1, 1.2, 1.3, 1.4, 1.5, 1.7, 1.8, 1.9, 2.0, 2.2, 2.3, 2.4, 2.5, 2.6, 2.8, 2.9, 3.0, 3.1, 3.3, 3.4, 3.5, 3.6, 3.7, 3.9, 4.0, 4.1, 4.2, 4.3, 4.5, 4.6, 4.7, 4.8, 5.0, 5.1, 5.2, 5.3, 5.4, 5.6, 5.7, 5.8, 5.9, 6.1, 6.2, 6.3, 6.4, 6.5, 6.7, 6.8, 6.9, 7.0, 7.1, 7.3, 7.4, 7.5, 7.6, 7.8, 7.9, 8.0, 8.1, 8.2, 8.4<br>*pulse length:* 100.0 ms<br>**full decays:**<br>*frequencies (kHz):* 10.0<br>*spin-lock durations (ms):* 1.0, 10.0, 20.0, 40.0, 60.0, 90.0, 140.0, 200.0 |

| | |
|---|---|
| $^{2}$H ubiquitin<br>600 MHz<br>55.5 kHz<br>non-decoupled | **cross polarisation**<br>*out:* $^{1}$H: 78.0 kHz (ramp90100.100), $^{15}$N: 31.0 kHz, 1.0 ms.<br>*back:* $^{1}$H: 78.0 kHz (ramp90100.100), $^{15}$N: 31.0 kHz, 1.0 ms.<br>**composite pulses**<br>*water suppression CPD:* 120.0 ms @ 16.0 kHz<br>*$^{1}$H decoupling*: waltz16, 25.0 µs @ 10.0 kHz<br>*$^{15}$N decoupling*: waltz16, 50.0 µs @ 5.0 kHz<br>**dimensions**<br>*$^{1}$H:* 16 scans, 29.7 ppm<br>*$^{15}$N:* 128 points, 40.0 ppm<br>**spin lock**<br>**fast mode:**<br>*frequencies (kHz):* 0.6, 0.8, 1.0, 1.1, 1.3, 1.5, 1.7, 1.8, 2.0, 2.2, 2.3, 2.5, 2.7, 2.8, 3.0, 3.2, 3.4, 3.5, 3.7, 3.9, 4.0, 4.2, 4.4, 4.6, 4.7, 4.9, 5.1, 5.2, 5.4, 5.6, 5.7, 5.9, 6.1, 6.3, 6.4, 6.6, 6.8, 6.9, 7.1, 7.3, 7.5, 7.6, 7.8, 8.0, 8.1, 8.3, 8.5, 8.6, 8.8, 9.0, 9.2, 9.3, 9.5, 9.7, 9.8, 10.0, 10.2, 10.4, 10.5, 10.7, 10.9, 11.0, 11.2, 11.4<br>*pulse length:* 100.0 ms<br>**full decays:**<br>*frequencies (kHz):* 4.2, 7.8, 11.4<br>*spin-lock durations (ms):* 1.0, 10.0, 20.0, 40.0, 60.0, 80.0, 110.0, 150.0 |
| $^{2}$H ubiquitin<br>700 MHz<br>100.0 kHz<br>non-decoupled | **cross polarisation**<br>*out:* $^{1}$H: 132.0 kHz (ramp90100.100), $^{15}$N: 27.0 kHz, 1.0 ms.<br>*back:* $^{1}$H: 132.0 kHz (ramp90100.100), $^{15}$N: 27.0 kHz, 1.0 ms.<br>**composite pulses**<br>*water suppression CPD:* 160.0 ms @ 10.0 kHz<br>*$^{1}$H decoupling*: waltz16, 25.0 µs @ 10.0 kHz<br>*$^{15}$N decoupling*: waltz16, 50.0 µs @ 5.0 kHz<br>**dimensions**<br>*$^{1}$H:* 8 scans, 29.8 ppm<br>*$^{15}$N:* 112 points, 33.0 ppm<br>**spin lock**<br>**fast mode:**<br>*frequencies (kHz):* 0.7, 0.8, 0.9, 1.1, 1.2, 1.3, 1.4, 1.6, 1.7, 1.8, 1.9, 2.0, 2.2, 2.3, 2.4, 2.5, 2.7, 2.8, 2.9, 3.0, 3.1, 3.3, 3.4, 3.5, 3.6, 3.8, 3.9, 4.0, 4.1, 4.2, 4.4, 4.5, 4.6, 4.7, 4.9, 5.0, 5.1, 5.2, 5.3, 5.5, 5.6, 5.7, 5.8, 6.0, 6.1, 6.2, 6.3, 6.4, 6.6, 6.7, 6.8, 6.9, 7.1, 7.2, 7.3, 7.4, 7.5, 7.7, 7.8, 7.9, 8.0, 8.2, 8.3, 8.4<br>*pulse length:* 100.0 ms<br>**full decays:**<br>*frequencies (kHz):* 1.8, 4.0, 5.1, 6.2, 7.3, 8.4<br>*spin-lock durations (ms):* 1.0, 10.0, 20.0, 40.0, 60.0, 80.0, 110.0, 150.0 |

| | |
|---|---|
| $^1$H ubiquitin<br>700 MHz<br>100.0 kHz<br>non-decoupled | **cross polarisation**<br>*out:* $^1$H: 129.0 kHz (ramp90100.100), $^{15}$N: 34.0 kHz, 1.0 ms.<br>*back:* $^1$H: 129.0 kHz (ramp90100.100), $^{15}$N: 34.0 kHz, 1.0 ms.<br>**composite pulses**<br>*water suppression CPD:* 160.0 ms @ 10.0 kHz<br>*$^1$H decoupling*: swftppm, 50.0 µs @ 10.0 kHz<br>*$^{15}$N decoupling*: waltz16, 50.0 µs @ 5.0 kHz<br>**dimensions**<br>*$^1$H:* 8 scans, 29.8 ppm<br>*$^{15}$N:* 112 points, 33.0 ppm<br>**spin lock**<br>**fast mode:**<br>*frequencies (kHz):* 0.7, 0.8, 0.9, 1.1, 1.2, 1.3, 1.4, 1.6, 1.7, 1.8, 1.9, 2.0, 2.2, 2.3, 2.4, 2.5, 2.7, 2.8, 2.9, 3.0, 3.1, 3.3, 3.4, 3.5, 3.6, 3.8, 3.9, 4.0, 4.1, 4.2, 4.4, 4.5, 4.6, 4.7, 4.9, 5.0, 5.1, 5.2, 5.3, 5.5, 5.6, 5.7, 5.8, 6.0, 6.1, 6.2, 6.3, 6.4, 6.6, 6.7, 6.8, 6.9, 7.1, 7.2, 7.3, 7.4, 7.5, 7.7, 7.8, 7.9, 8.0, 8.2, 8.3, 8.4<br>*pulse length:* 100.0 ms<br>**full decays:**<br>*frequencies (kHz):* 1.8, 4.0, 5.1, 6.2, 7.3<br>*spin-lock durations (ms):* 1.0, 10.0, 20.0, 40.0, 60.0, 80.0, 110.0, 150.0 |
| $^1$H ubiquitin<br>700 MHz<br>100.0 kHz<br>CW-decoupled | **cross polarisation**<br>*out:* $^1$H: 130.0 kHz (ramp90100.100), $^{15}$N: 32.0 kHz, 1.0 ms.<br>*back:* $^1$H: 130.0 kHz (ramp10090.100), $^{15}$N: 32.0 kHz, 1.0 ms.<br>**composite pulses**<br>*water suppression CPD:* 40.0 ms @ 10.0 kHz<br>*$^1$H decoupling*: swftppm, 50.0 µs @ 10.0 kHz<br>*$^{15}$N decoupling*: waltz16, 50.0 µs @ 5.0 kHz<br>**dimensions**<br>*$^1$H:* 16 scans, 29.8 ppm<br>*$^{15}$N:* 128 points, 33.0 ppm<br>**spin lock**<br>**fast mode:**<br>*frequencies (kHz):* 0.7, 0.8, 0.9, 1.1, 1.2, 1.3, 1.4, 1.6, 1.7, 1.8, 1.9, 2.0, 2.2, 2.3, 2.4, 2.5, 2.7, 2.8, 2.9, 3.0, 3.1, 3.3, 3.4, 3.5, 3.6, 3.8, 3.9, 4.0, 4.1, 4.2, 4.4, 4.5, 4.6, 4.7, 4.9, 5.0, 5.1, 5.2, 5.3, 5.5, 5.6, 5.7, 5.8, 6.0, 6.1, 6.2, 6.3, 6.4, 6.6, 6.7, 6.8, 6.9, 7.1, 7.2, 7.3, 7.4, 7.5, 7.7, 7.8, 7.9, 8.0, 8.2, 8.3, 8.4<br>*pulse length:* 40.0 ms<br>**full decays:**<br>*frequencies (kHz):* 1.8, 4.0, 6.2, 8.4<br>*spin-lock durations (ms):* 1.0, 10.0, 20.0, 30.0, 40.0, 60.0, 80.0, 100.0 |

| | |
|---|---|
| $^{2}H$ ubiquitin<br>700 MHz<br>100.0 kHz<br>CW-decoupled | **cross polarisation**<br>*out:* $^{1}H$: 130.0 kHz (ramp90100.100), $^{15}N$: 32.0 kHz, 1.0 ms.<br>*back:* $^{1}H$: 130.0 kHz (ramp10090.100), $^{15}N$: 32.0 kHz, 1.0 ms.<br>**composite pulses**<br>*water suppression CPD:* 40.0 ms @ 10.0 kHz<br>*$^{1}H$ decoupling*: swftppm, 50.0 µs @ 10.0 kHz<br>*$^{15}N$ decoupling*: waltz16, 50.0 µs @ 5.0 kHz<br>**dimensions**<br>*$^{1}H$:* 16 scans, 29.8 ppm<br>*$^{15}N$:* 128 points, 33.0 ppm<br>**spin lock**<br>**fast mode:**<br>*frequencies (kHz):* 0.7, 0.8, 0.9, 1.1, 1.2, 1.3, 1.4, 1.6, 1.7, 1.8, 1.9, 2.0, 2.2, 2.3, 2.4, 2.5, 2.7, 2.8, 2.9, 3.0, 3.1, 3.3, 3.4, 3.5, 3.6, 3.8, 3.9, 4.0, 4.1, 4.2, 4.4, 4.5, 4.6, 4.7, 4.9, 5.0, 5.1, 5.2, 5.3, 5.5, 5.6, 5.7, 5.8, 6.0, 6.1, 6.2, 6.3, 6.4, 6.6, 6.7, 6.8, 6.9, 7.1, 7.2, 7.3, 7.4, 7.5, 7.7, 7.8, 7.9, 8.0, 8.2, 8.3, 8.4<br>*pulse length:* 40.0 ms<br>**full decays:**<br>*frequencies (kHz):* 1.8, 4.0, 6.2, 8.4<br>*spin-lock durations (ms):* 1.0, 10.0, 20.0, 30.0, 40.0, 60.0, 80.0, 100.0 |

## 2 Validity of Single Point Relaxation Measurement in the Solid State

An assumption inherent in the single-point method for measuring dispersion curves outlined in the main text is that the decay of the magnetisation under spin-lock in an $R_{1\rho}$ experiment is effectively mono-exponential. However, it is well-known that this decay is inherently multi-exponential because the rate of dipolar- and CSA-induced relaxation depends on the orientation relative to the static magnetic field [1, 2].

For the purposes of the investigation here we are less interested in the exact magnitude of the $R_{1\rho}$ and more interested in the change of $R_{1\rho}$ as a function of the RF field strength, and in an increased frequency resolution in this dispersion profile. Indeed, given the prevalence of the 'bump' artefacts in the non-decoupled datasets here, it is questionable how much relevance the actual fit $R_{1\rho}$ has in those cases. Consequently, we would argue that for the majority of the datasets here, it was more important to obtain the frequency resolution allowed by the single-point method, even if that imposed a significant additional error on the measured $R_{1\rho}$. However, for the purposes of the quantitation of dynamics from the decoupled curves it is important to understand what potential biases or errors exist that analysing the data in this manner may have.

The BMRD contribution to the relaxation rate constant of interest here arises due to the stochastic variation in the isotropic chemical shift. It is therefore not expected to vary for different orientations within the powder pattern. The intensity of $x$ spin magnetisation in a multiexponential manner at time $t$ is thus:

$$I(\nu_1;t) = I(0)\sum_n c_n \exp(-(R_{1\rho}^{aniso}(\Omega_n,\nu_1) + R_{1\rho}^{iso}(\nu_1))t) \tag{1}$$

$$= I(0)\exp(-R_{1\rho}^{iso}(\nu_1)t)\sum_n c_n \exp(-R_{1\rho}^{aniso}(\Omega_n,\nu_1)t) \tag{2}$$

where the sum is over different powder orientations $\Omega_n$, each with a weighting $c_n$. Expressions for these different orientation dependencies may be found in the literature [1, 3]; for the purposes of the discussion here, the exact form is unimportant. Following on from equation 9 in the main text (reproduced here), we calculate our $R_{1\rho}$ from the intensity measured at a constant time:

$$\left[R_{1\rho}(\nu_1) + \text{offset}\right] = -\frac{1}{t}\ln\left(I(\nu_1;t)\right). \tag{3}$$

If we apply the right hand side of this equation to the intensity given in equation 1, we obtain

$$-\frac{1}{t}\ln\left(I(\nu_1;t)\right) = -\frac{1}{t}\ln\left(I(0)\exp(-R_{1\rho}^{iso}(\nu_1)t)\sum_n c_n \exp(-R_{1\rho}^{aniso}(\Omega_n,\nu_1)t)\right) \tag{4}$$

$$= -\frac{1}{t}\ln\left(I(0)\right) - \frac{1}{t}\ln\left(\sum_n c_n \exp(-R_{1\rho}^{aniso}(\Omega_n,\nu_1)t)\right) + R_{1\rho}^{iso}(\nu_1). \tag{5}$$

By comparison of equations 3 and 5 it may be seen that equation 3 is only strictly valid for this kind of multiexponential decay if we may assume that $\sum_n c_n \exp(-R_{1\rho}^{aniso}(\Omega_n,\nu_1)t)$ is independent of $\nu_1$. However, equation 3 is a reasonable approximation so long as the variation in the anisotropic contribution is small across the range of frequencies which we are interested in.

The largest contribution to a $\nu_1$ dependency in the anisotropic component of $R_{1\rho}$ for an amide N-H bond arises due to the low frequency spectral density terms comprising the heteronuclear dipolar interaction[4]. These may be written [3]:

$$R_{1\rho}^{aniso}(\nu_1,\nu_r) = \sum_n c_n \exp(-R_{1\rho}^{aniso}(\Omega_n,\nu_1)t) \tag{6}$$

$$= \frac{C_{NH}^2}{60}\left[2J(\nu_1-2\nu_r)+4J(\nu_1-\nu_r)+4J(\nu_1+\nu_r)+2J(\nu_1+2\nu_r)\right] \tag{7}$$

$$J(\nu) = \frac{(1-S^2)\tau}{1+(2\pi\nu\tau)^2} \tag{8}$$

where $C_{NH}^2$ is the squared dipolar coupling constant ($C_{NH} = -\frac{\mu_0}{4\pi}\frac{\gamma_N\gamma_H\hbar}{\langle r_{NH}^3\rangle}$, where $\mu_0$ is the vacuum permeability). We are then interested in the difference between $R_{1\rho}^{aniso}(\nu_1,\nu_r)$ at the upper and lower ends of our measured dispersion:

$$\Delta R_{1\rho}^{aniso}(\nu_1^A,\nu_1^B,\nu_r) = R_{1\rho}^{aniso}(\nu_1^A,\nu_r) - R_{1\rho}^{aniso}(\nu_1^B,\nu_r). \tag{9}$$

For representative conditions for the experiments measured here, we take $\nu_1^A$ =8.4 kHz, $\nu_1^B$ =1 kHz at $\nu_r$ =55.55 kHz. Differentiating equation 9 with respect to $\tau$, we find that the timescale to which the difference is most sensitive is $\tau$ =6.9 × $10^{-6}$ srad$^{-1}$ (4.3 × $10^{-5}$ s). For this timescale, we find that:

$$\Delta R_{1\rho}^{aniso}(\nu_1^A,\nu_1^B,\nu_r) = 34.79(1-S^2). \tag{10}$$

Given our experimental error, it is reasonable to assume that the assumption of an effective $\nu_1$ independent offset value is valid so long as $\Delta R_{1\rho}$ <1 s$^{-1}$. From equation 10, this is the case under these assumptions so long as $S^2 > 0.971$. This is in excess of the majority of previously reported µs order parameters for the ubiquitin and similar systems [5, 6, 7] (with a few notable exceptions, such as residue 54 in ubiquitin in Reference [8]), and thus we would not expect any significant spin lock variation in the anisotropic component of the decay, and consequently the assumptions underpinning the use of a single point analysis in this case are valid.

To further validate this experimentally, we made use of the datasets recorded in a typical time series manner for 100%-$^1$H backexchanged $^2$H, $^{13}$C, $^{15}$N TET2 at 55 kHz, both with and without 16 kHz CW $^1$H decoupling. In the decoupled case, we recorded five relaxation decays at applied nutation frequencies of (1.9 kHz, 3.1 kHz, 4.2 kHz, 6.6 kHz, 8.9 kHz) using eight time points (1 ms, 10 ms, 20 ms, 40 ms, 60 ms, 80 ms, 110 ms, 150 ms). In the non-decoupled case, we recorded relaxation decays at eleven applied nutation frequencies (0.7 kHz, 1.4 kHz, 2.1 kHz, 2.8 kHz, 3.5 kHz, 4.2 kHz, 4.9 kHz, 5.6 kHz, 6.3 kHz, 7.0 kHz, 7.7 kHz, 8.4 kHz) at six time points (2 ms, 5 ms, 10 ms, 25 ms, 50 ms, 100 ms).

For the analysis, we took the same set of decay curves (TET2, 55.55 kHz, decoupled and non-decoupled, separately), and investigated how the $R_{1\rho}$ rates measured in a single-point manner compared to those measured in a multi-point manner. Specifically, for each residue and experimental setup we took the set of decay curves recorded at different nutation frequencies. The multi-point relaxation rate was determined by fitting a single mono-exponential to each decay (see main text - note that there was no significant evidence for multi-exponentiality in these decay curves, with Akaike Information Criterion selecting a mono-exponential model over a bi-exponential model in almost all cases). To determine the single-point equivalent, we performed the analysis detailed in the main text to determine $\left[R'_{1\rho}(\nu_1) + \text{offset}\right]$ for the intensity and noise level measured at the closest time to 100 ms (100 ms for the non-decoupled case, 110 ms for the decoupled case). These were then corrected to $R'_{1\rho}(\nu_1)$ using the median offset between the datasets, analogously to the method outlined in the main text.

Figure S2 shows some representative examples of this analysis. In the top curves for each residual, BMRD dispersion curves are shown as measured using the bi/mono-exponential analysis on the full time decay curves (blue) and the single-point analysis (red). We additionally show the deviation of the offset determined for each spin-lock field strength, offset($\nu_1$), with the median offset. For the vast

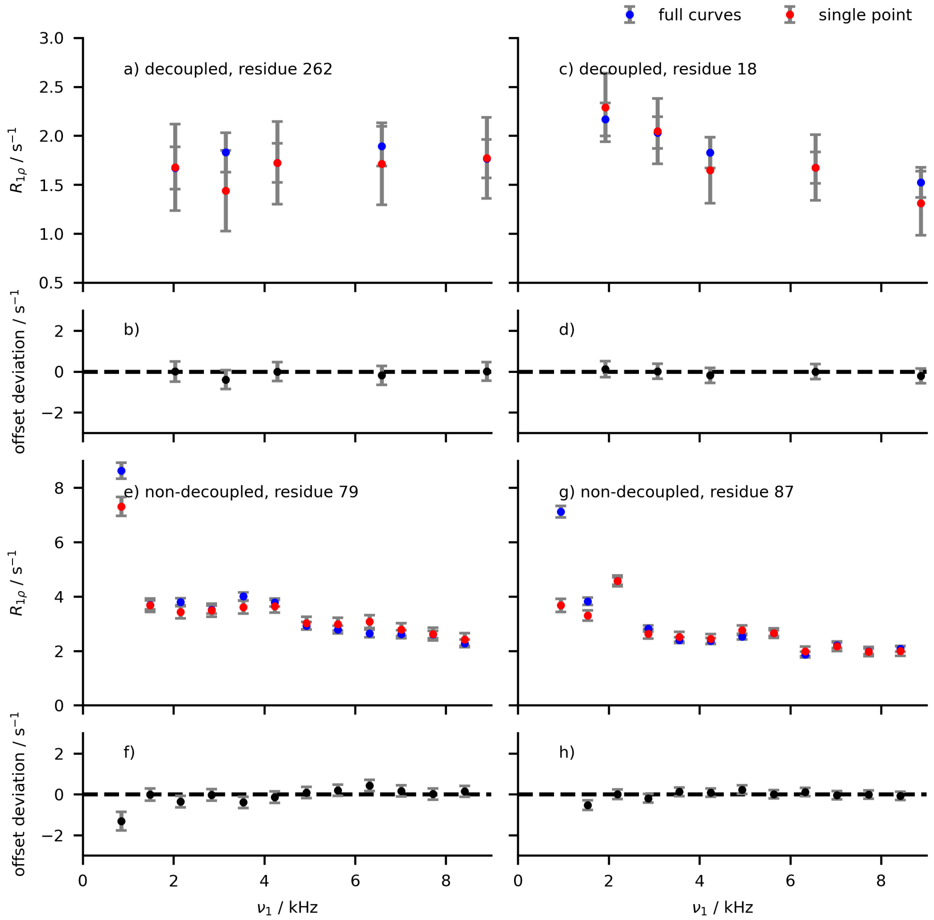


**Figure S2:** Comparison of dispersion curves (a,c,e,g) determined using either model fitting to a time decay curve (blue) or a single-point measurement (red). The nutation frequency specific deviation in offset from the median offset used in the correction is shown in (b,d,f,h).

majority of cases, the $R_{1\rho}(\nu_1)$ determined in each way are within uncertainty of one another (see the main text for details on uncertainty estimation). The largest deviations tend to occur in the lowest frequencies (<2 kHz), which likely occurs due to the spin-spin coherent decay becoming significant under these conditions and leading to significant oscillations in the decay curves (e.g., the assumption that the anisotropic part is approximately independent of $\nu_1$ breaks down).

Put together, these analyses suggest that the assumptions inherent in the single-point analysis used here are valid. While the assumption of mono-exponentiality in the analysis is a significant approximation, it gives reasonable values of $R_{1\rho}$ without incurring significant deviation from a more complete analysis. We also note that this assumption is likely less impactful than other assumptions which are made in the further analysis of $R_{1\rho}$, such as the typical assumptions of two-site exchange or a single exchange rate.

# 3 Alternate Decoupling Schemes

We additionally tried using Two-Pulse Phase-Modulated (TPPM) [9] decoupling for removing the bump artefacts. Some of our trial experiments are shown in Figure S3. These were performed in a pseudo-2D manner, without site-specific resolution. The offset correction described in the main text was not performed, as we did not attempt to record full $R_{1\rho}$ decay curves to allow for this. In these experiments, the TPPM angle was set to 15°. While TPPM removed the artefact, the large number of combinations between the modulation frequency and the effective-field frequency show up as several new detrimental first-order and second-order recoupling conditions arising in the profiles [10]. A detailed analysis is beyond the scope of this publication but a rough estimate showed that the strong recoupling condition around 5 kHz for a pulse length of 10 µs corresponds to a match of the modulation frequency of the TPPM and the spin-lock frequency to the MAS frequency which leads to a first-order recoupling of the heteronuclear dipolar coupling. The other recoupling conditions are most likely second-order recoupling conditions due to their much lower intensity.

# 4 Modelling the Bumps by Consideration of the Local Chemical Environment

As the 'bump' artefacts arise from irradiation of the $^{15}N$ spins at the chemical shift difference of two $^{1}H$ spins, and given that we have arrived at a method which can remove this additional contribution, an immediate thought which arises is whether the difference between the decoupled and non-decoupled BMRD profiles might itself provide insight into the local proton environment around a given amide site.

To investigate this further, we took all of the 100 kHz non- and CW- decoupled BMRD profiles recorded on protonated ubiquitin. We calculated the 'bump residual', which we term here $\Delta_{1\rho}$, as:

$$\Delta_{1\rho} = R_{1\rho}^{\text{no-decoupling}} - R_{1\rho}^{CW}. \tag{11}$$

In performing the following analysis, we were careful to omit any sites showing significant overlap, as this would lead to complications in the analysis. We assume that one of the $^{1}H$ spins contributing to the bumps in each case is the amide proton, and that all other contributing sites are at lower chemical shifts than this amide proton. This enables us to convert the BMRD spin-lock irradiation frequency into a quasi-chemical shift, $\delta$ with units of ppm, as:

$$\delta = \delta_{\text{amide,H}} - \frac{\nu_1}{\nu_{0,H} \times 10^{-6}}, \tag{12}$$

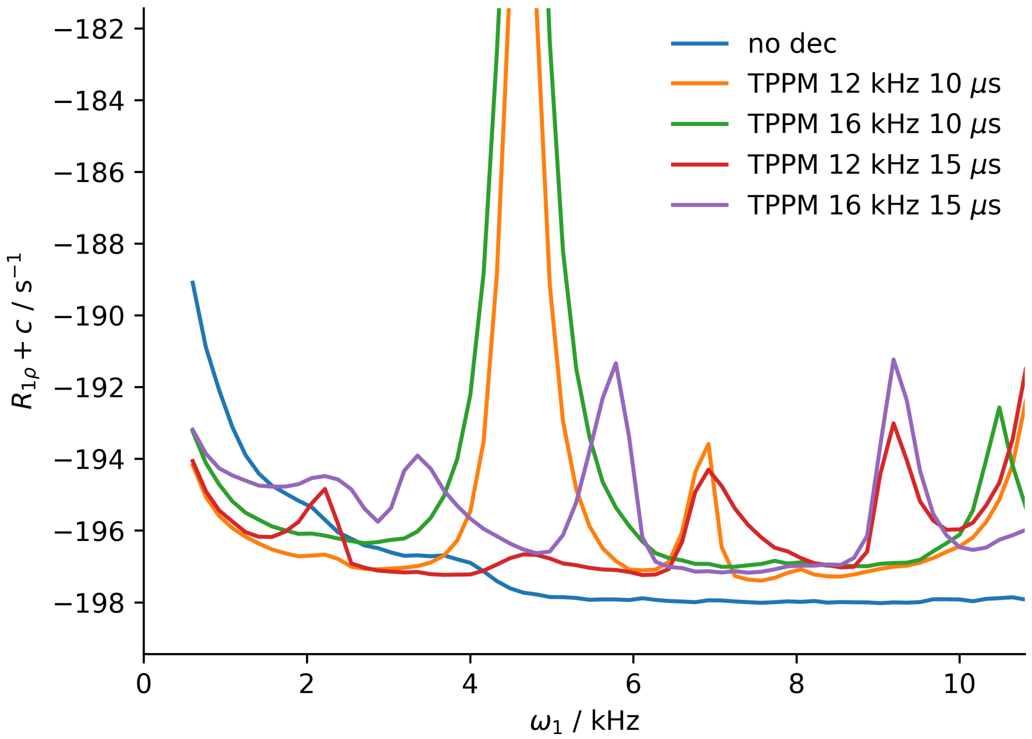


**Figure S3:** Trial experiments with TPPM decoupling. The offset $R'_{1\rho}$ values were determined through the method described in the Methods section of the main text, up to equations 8 and 9 (noting that we did not measure full decay curves in this case).

where $\delta_{\text{amide,H}}$ is the amide proton chemical shift, $\nu_1$ is the applied spin-lock field strength in units of Hz, and $\nu_{0,H}$ is the $^1$H Larmor frequency in units of Hz. For modelling the bumps, we introduce a phenomenological model:

$$\Delta_{1\rho}^{\text{model}}(\delta) = A \sum_{i=1}^{200} \frac{1}{r_i^a} \exp\left(-\frac{(\delta_{\text{amide,H}} - B \times |\delta_i - \delta_{\text{amide,H}}| - \delta)^2}{C}\right), \tag{13}$$

where the summation is over the 200 closest protons to the amide proton (indexed using $i$), $\delta_i$ is the chemical shift (in ppm) of the $i$th proton, $r_i$ is the distance between the $i$th proton and the amide proton, and $A$, $B$, $C$, $a$ are fit parameters relating to the amplitude, MAS-dependent frequency scaling (see Figure 2c,d, and surrounding discussion), width, and distance scaling, respectively. In this analysis, we obtained $^1$H positions by using 'addh' in ChimeraX[11] on the 1UBQ ubiquitin structure,[12] and took backbone and sidechain $^1$H chemical shifts from Weber *et al.*(1987)[13]. It should be noted that the coverage of these chemical shifts is not comprehensive, however the sites missing shifts are typically those further along the side chains and thus less likely to contribute to the artefacts. While these chemical shifts were measured in solution state and thus may not be precisely the same in the microcrystal, we are limited here to at most a resolution of 0.17 ppm and so the precision of the chemical shifts used for modeling is unlikely to be the greatest contributor to uncertainty in this analysis.

All good quality (e.g., non-overlapping, fitting artefact free) $\Delta_{1\rho}(\delta)$ profiles were fit simultaneously using the 'minimize' routine in SciPy.[14] The resulting fit parameters (with uncertainties estimated at one standard deviation using the inverse of the Hessian returned by 'minimize') were: $A = (107.6 \pm 0.1)\ \text{s}^{-1}$, $B = (0.902 \pm 0.001)$, $C = (0.62 \pm 0.02)\ \text{ppm}^2$, $a = (3.72 \pm 0.01)$. The resulting profiles are shown in purple in Figure S4.

In addition, we performed a comparison for the case of a deuterated protein. We took the same resulting fit parameters as determined from the $^1$H case, but removed any hydrogen sites that would be expected to carry a $^2$H nucleus in this system (aside from the site-specific labeling of the two δsites

with $^1$H in the arginines present in this specifically labeled sample, we omitted any $^1$H not bonded to nitrogen or oxygen - note however, that the proportion of the sites retained which do not have assignments is increased). These profiles are shown in green in Figure S4.
We find that in general, there is rather good agreement between the modelled profiles and the experimental $\Delta_{1\rho}$ curves in the $^1$H case. The agreement in the $^2$H ubiquitin is generally less good, but in many cases the model is still generates features similar to those found experimentally (for instance, the 4 ppm bump in residue 36, or the 6 ppm bumps in residues 33 and 59) - this is remarkable since no additional fitting was performed in the $^2$H case.

## 5 Using Alignment Pulses Before/After Spin Lock

In the experiments described in the main text, no alignment pulses were used, and as such the magnetisation at the start of the spin-lock pulse was aligned along the $x$ axis in the transverse plane. While we accounted for this offset analytically (equation 6 in the main paper), we additionally performed verification experiments using pulses to align the initial magnetisation along the axis of the spin-lock. We performed experiments equivalent to those outlined in the Methods section, with the addition of tangential alignment pulses before and after the spin-lock[15]. These showed no significant deviations from the experiments without the spin-lock pulses (see Figure S5). In the main experiments, we chose not to include these pulses, as it removed the necessity to model how the balance between $R_1$ and $R_{1\rho}$ contributions varies during the 8 ms during which the magnetisation is being aligned.

## 6 Representative GAMMA Simulation Input Files

Below, representative spin system definition files and commands to run the GAMMA simulations (see Figure 3) are given. The simulation source code is given in the Supplementary Materials.

```
spin.sys
---
SysName (2) : SpinSystem
NSpins (0): 3
Iso(0) (2): 15N
Iso(1) (2): 1H
Iso(2) (2): 1H
Omega (1): 700
```

```
run_single.sh
---
./bump_sim spin.sys \
            0.000000 22954.831832 180.000000 90.000000 0.000000 \
            0.000000 8661.181422 -120.198002 90.000000 0.000000 \
            0.000000 120450.147606 -75.742965 90.000000 0.000000 \
            0 0 0 0 0 0 0 \
            700 0 0 0 0 0 30 \
            -700 0 0 0 0 0 30 \
            2 50 $1 100 55555 10 sim_${1}_55555
```

This corresponds to the following interactions:

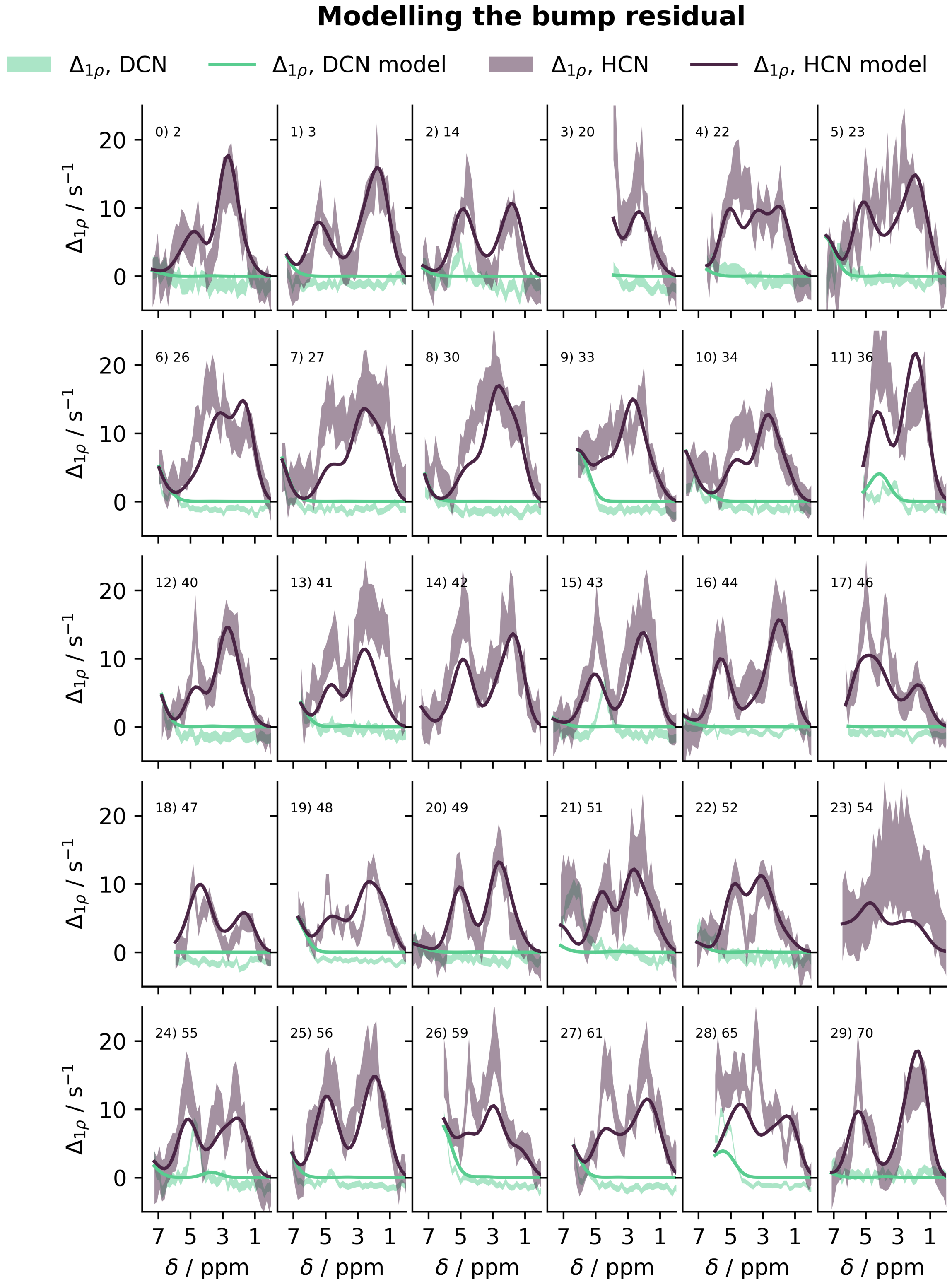


**Figure S4:** Four parameter model fits to all high-quality well-resolved $\Delta_{1\rho}$ profiles recorded on $^{1}$H ubiquitin at 100 kHz MAS (purple). The resulting model was then applied to the perdeuterated case without further fitting (green).

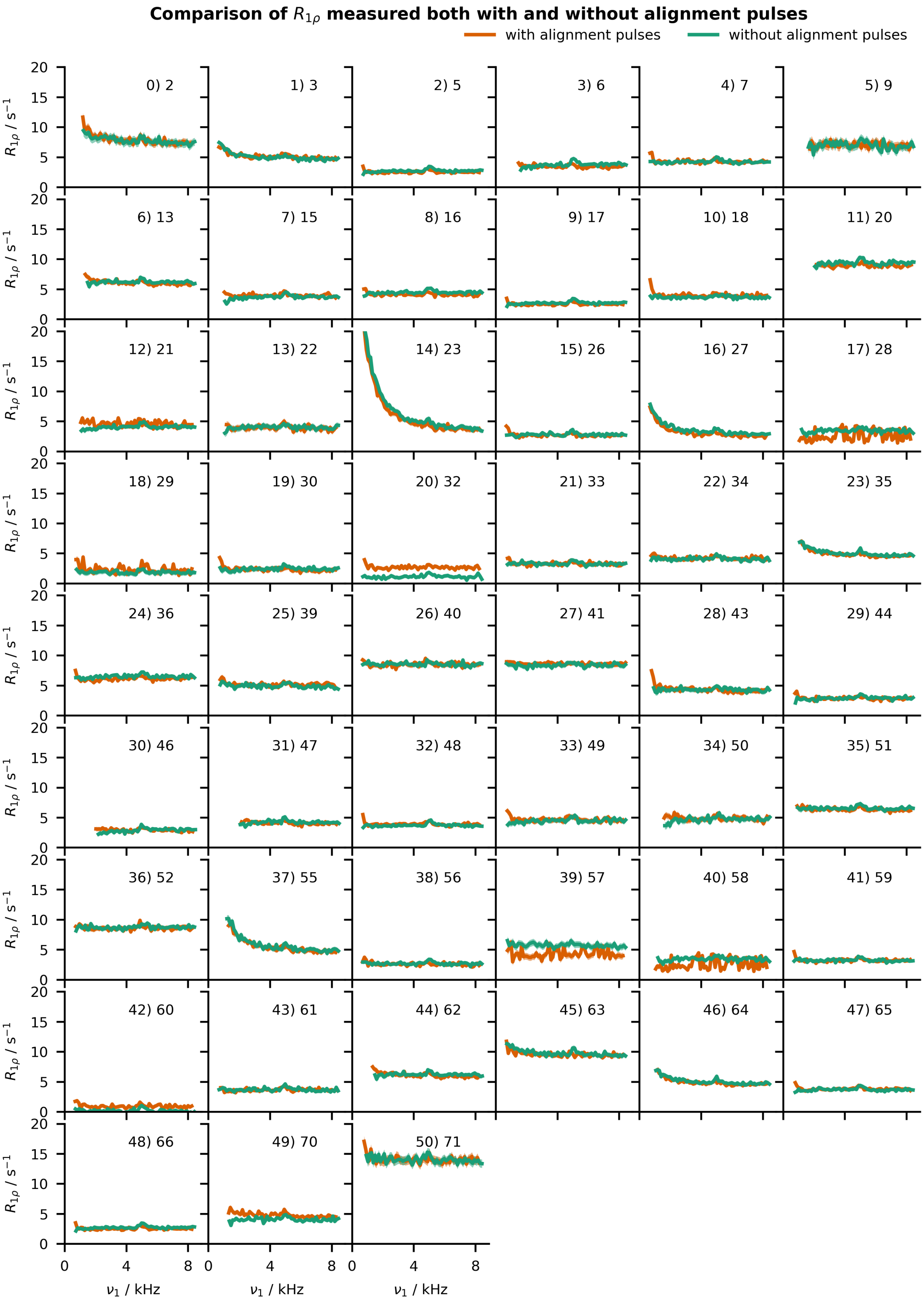


**Figure S5:** Comparison of BMRD profiles recorded with and without alignment pulses before and after the spin-lock. Measured on $^2$H ubiquitin at 700 MHz $\nu_0^H$ and 100 kHz MAS.

| type | spins | magnitude / Hz | $\alpha$ / ° | $\beta$ / ° | $\gamma$ / ° |
|---|---|---|---|---|---|
| dipole | N, H1 | 11477.42 | 180.0 | 90.0 | 0.0 |
| dipole | N, H2 | 4330.59 | -120.2 | 90.0 | 0.0 |
| dipole | H1, H2 | 60225.07 | -75.7 | 90.0 | 0.0 |
| shift | N | 0.00 | 0.0 | 0.0 | 0.0 |
| shift | H1 | 700.00 | 0.0 | 0.0 | 0.0 |
| shift | H2 | -700.00 | 0.0 | 0.0 | 0.0 |

Note that GAMMA simulations take the anisotropy of the dipolar coupling, which is twice the dipolar coupling.

```
run_many.sh
---
seq 0 5 2000 | parallel -j6 --ungroup ./run_single.sh {}
```

## 7 Potential for bump-like artefacts in NERRD profiles

While we have paid most attention here to the effect of these artefacts on BMRD dispersion profiles, the MIRROR recoupling condition may additionally pose challenges for NERRD measurements. In particular, the $n = 1$ condition in equation 1 could be met for spin-lock fields approaching the MAS frequency. Figure S6 shows simulations of such an effect (where the chemical shift difference of the adjacent protons was set to 4 kHz). While the profile is dominated by the $\nu_1 = \nu_r$ rotary resonance recoupling ($R^3$) condition at 55.56 kHz (note that we have omitted the region immediately around this condition, as the simulation parameters we are using are unsuitable for adequate quantitation of the relaxation in this region), 'bumps' additionally appear at 51.56 kHz and 59.56 kHz owing to the MIRROR condition.
The application of CW decoupling to remove these would likely be more challenging than in the BMRD case, as these would affect both the sampling of the spectral density functions composing the incoherent part of the dispersion, and would additionally affect the coherent spin Hamiltonian. Further, the assumptions that underpin the application of the single-point method used here would not be applicable in the case of NERRD, where the relaxation is known to be highly multi-exponential.

## References


[1] N. Giraud, M. Blackledge, M. Goldman, A. Böckmann, A. Lesage, F. Penin, L. Emsley, *J. Am. Chem Soc.* **2005**, *127*, 18190.

[2] P. Schanda, M. Ernst, *Prog. Nucl. Magn. Reson. Spectrosc.* **2016**, *96*, 1.

[3] A. Krushelnitsky, G. Hempel, H. Jurack, T. M. Ferreira, *Phys. Chem. Chem. Phys.* **2023**, *25*, 15885.

[4] J. Sein, N. Giraud, M. Blackledge, L. Emsley, *Journal of Magnetic Resonance* **2007**, *186*, 26.

[5] P. Ma, Y. Xue, N. Coquelle, J. D. Haller, T. Yuwen, I. Ayala, O. Mikhailovskii, D. Willbold, J.-P. Colletier, N. R. Skrynnikov, P. Schanda, *Nature Communications* **2015**, *6*, 8361.

[6] V. Kurauskas, S. A. Izmailov, O. N. Rogacheva, A. Hessel, I. Ayala, J. Woodhouse, A. Shilova, Y. Xue, T. Yuwen, N. Coquelle, J.-p. Colletier, N. R. Skrynnikov, P. Schanda, *Nat. Commun.* **2017**, *8*, 145.

[7] A. Krushelnitsky, D. Gauto, D. C. Rodriguez Camargo, P. Schanda, K. Saalwächter, *Journal of Biomolecular NMR* **2018**, *71*, 53.

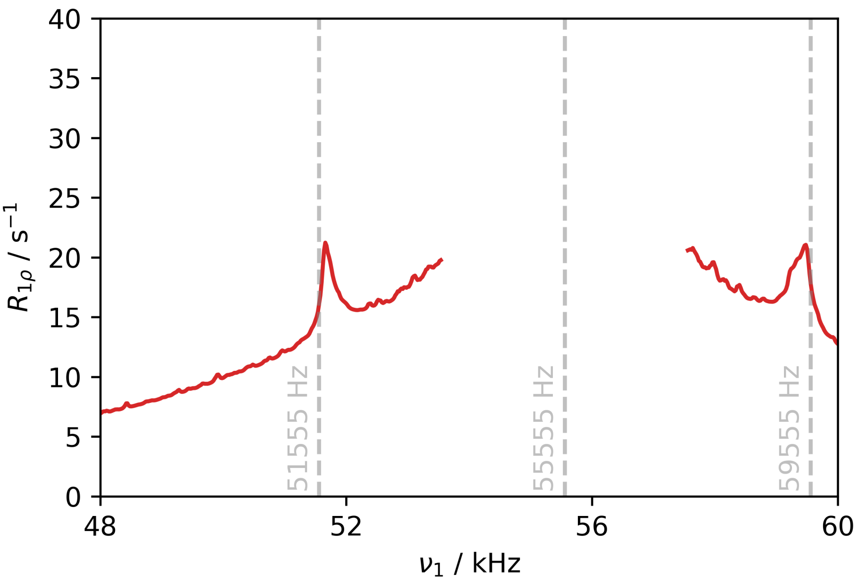


**Figure S6:** Simulation of 'bumps' arising in the NERRD regime. The spin system was set up as in Figure 3, with the chemical shift difference adjusted to 4 kHz to better separate this from the $R^3$ recoupling condition. The MAS frequency was set to 55.56 kHz.


[8] P. Ma, J. D. Haller, J. Zajakala, P. Macek, A. C. Sivertsen, D. Willbold, J. Boisbouvier, P. Schanda, *Angewandte Chemie International Edition* **2014**, *53*, 4312.

[9] A. E. Bennett, C. M. Rienstra, M. Auger, K. V. Lakshmi, R. G. Griffin, *The Journal of Chemical Physics* **1995**, *103*, 6951.

[10] I. Scholz, P. Hodgkinson, B. H. Meier, M. Ernst, *The Journal of Chemical Physics* **2009**, *130*, 114510.

[11] T. D. Goddard, C. C. Huang, E. C. Meng, E. F. Pettersen, G. S. Couch, J. H. Morris, T. E. Ferrin, *Protein Science* **2018**, *27*, 14.

[12] S. Vijay-Kumar, C. E. Bugg, W. J. Cook, *Journal of Molecular Biology* **1987**, *194*, 531.

[13] P. L. Weber, S. C. Brown, L. Mueller, *Biochemistry* **1987**, *26*, 7282, pMID: 2827749.

[14] P. Virtanen, R. Gommers, T. E. Oliphant, M. Haberland, T. Reddy, D. Cournapeau, E. Burovski, P. Peterson, W. Weckesser, J. Bright, S. J. van der Walt, M. Brett, J. Wilson, K. J. Millman, N. Mayorov, A. R. J. Nelson, E. Jones, R. Kern, E. Larson, C. J. Carey, İ. Polat, Y. Feng, E. W. Moore, J. VanderPlas, D. Laxalde, J. Perktold, R. Cimrman, I. Henriksen, E. A. Quintero, C. R. Harris, A. M. Archibald, A. H. Ribeiro, F. Pedregosa, P. van Mulbregt, SciPy 1.0 Contributors, A. Vijaykumar, A. P. Bardelli, A. Rothberg, A. Hilboll, A. Kloeckner, A. Scopatz, A. Lee, A. Rokem, C. N. Woods, C. Fulton, C. Masson, C. Häggström, C. Fitzgerald, D. A. Nicholson, D. R. Hagen, D. V. Pasechnik, E. Olivetti, E. Martin, E. Wieser, F. Silva, F. Lenders, F. Wilhelm, G. Young, G. A. Price, G.-L. Ingold, G. E. Allen, G. R. Lee, H. Audren, I. Probst, J. P. Dietrich, J. Silterra, J. T. Webber, J. Slavič, J. Nothman, J. Buchner, J. Kulick, J. L. Schönberger, J. V. de Miranda Cardoso, J. Reimer, J. Harrington, J. L. C. Rodríguez, J. Nunez-Iglesias, J. Kuczynski, K. Tritz, M. Thoma, M. Newville, M. Kümmerer, M. Bolingbroke, M. Tartre, M. Pak, N. J. Smith, N. Nowaczyk, N. Shebanov, O. Pavlyk, P. A. Brodtkorb, P. Lee, R. T. McGibbon, R. Feldbauer, S. Lewis, S. Tygier, S. Sievert, S. Vigna, S. Peterson, S. More, T. Pudlik, T. Oshima, T. J. Pingel, T. P. Robitaille, T. Spura, T. R. Jones, T. Cera, T. Leslie, T. Zito, T. Krauss, U. Upadhyay, Y. O. Halchenko, Y. Vázquez-Baeza, *Nature Methods* **2020**, *17*, 261.

[15] F. A. Mulder, R. A. de Graaf, R. Kaptein, R. Boelens, *Journal of Magnetic Resonance* **1998**, *131*, 351.

# 8 All Dispersion Curves

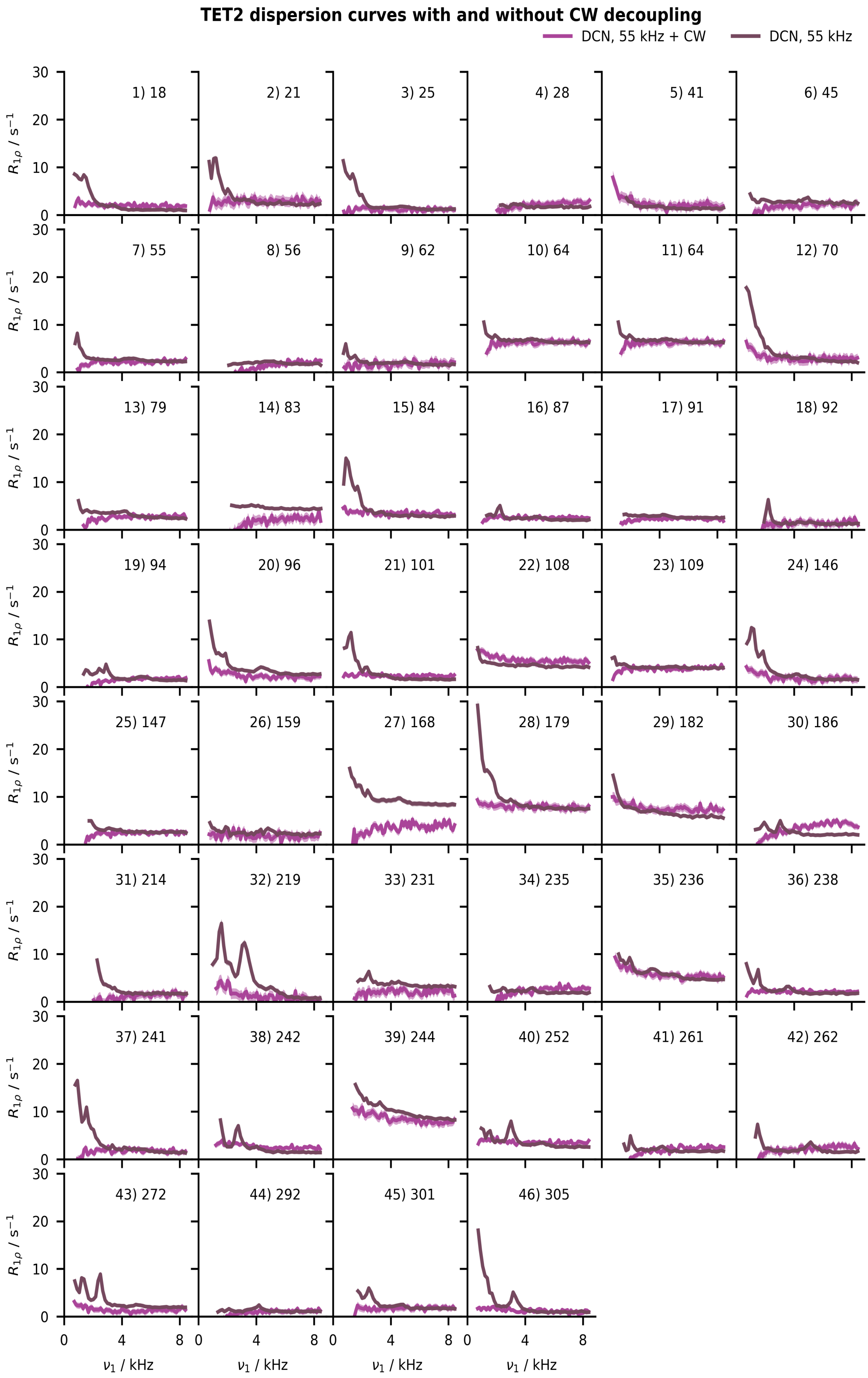


**Figure S7:** All dispersion curves measured for perdeuterated TET2 at a MAS rate of 55.55 kHz and 700 MHz $\nu_0^H$, both with and without 16 kHz CW decoupling.

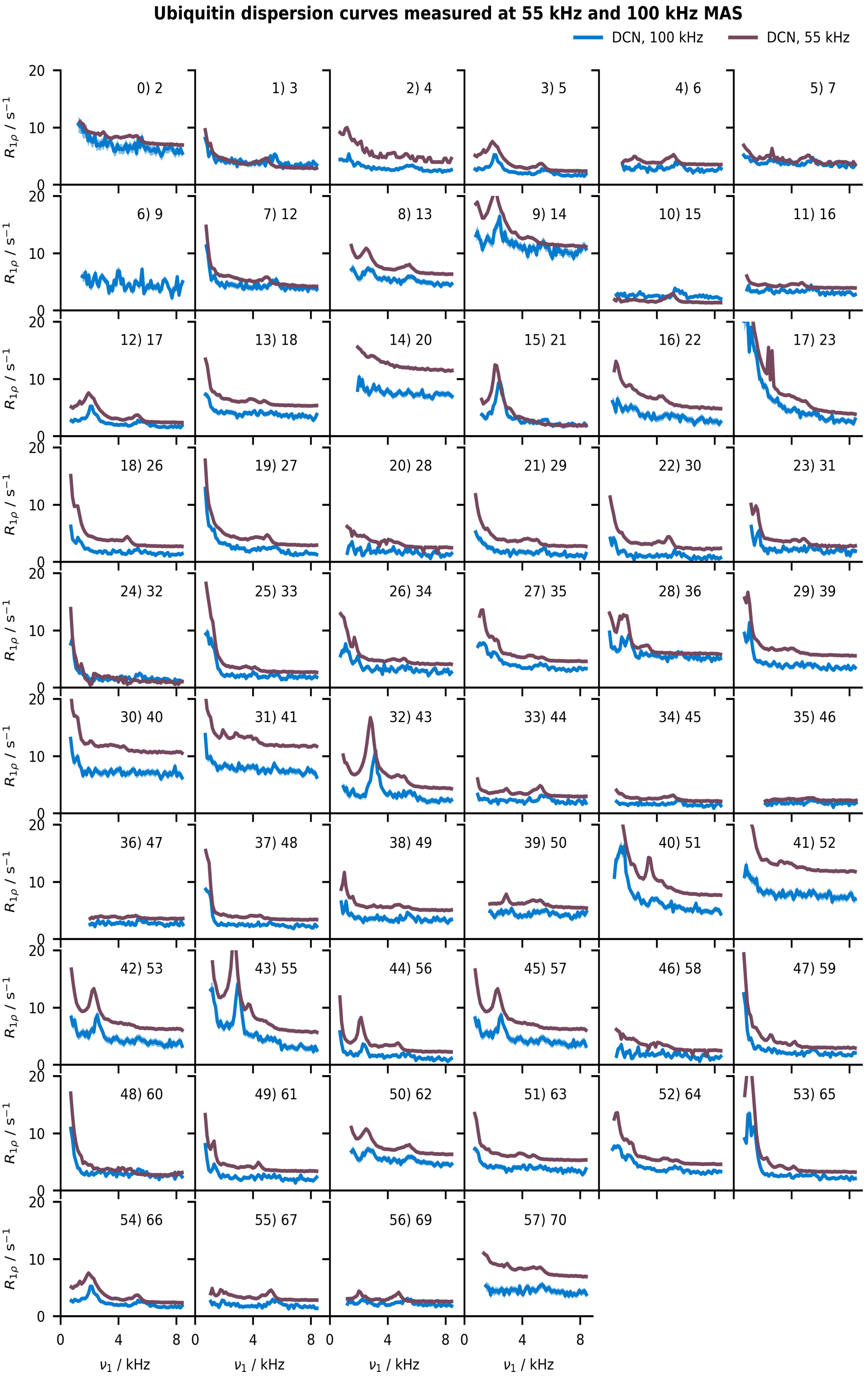


**Figure S8:** All dispersion curves measured for perdeuterated ubiquitin at 700 MHz $^1$H Larmor frequency, at MAS rates of 55.55 kHz and 100 kHz.

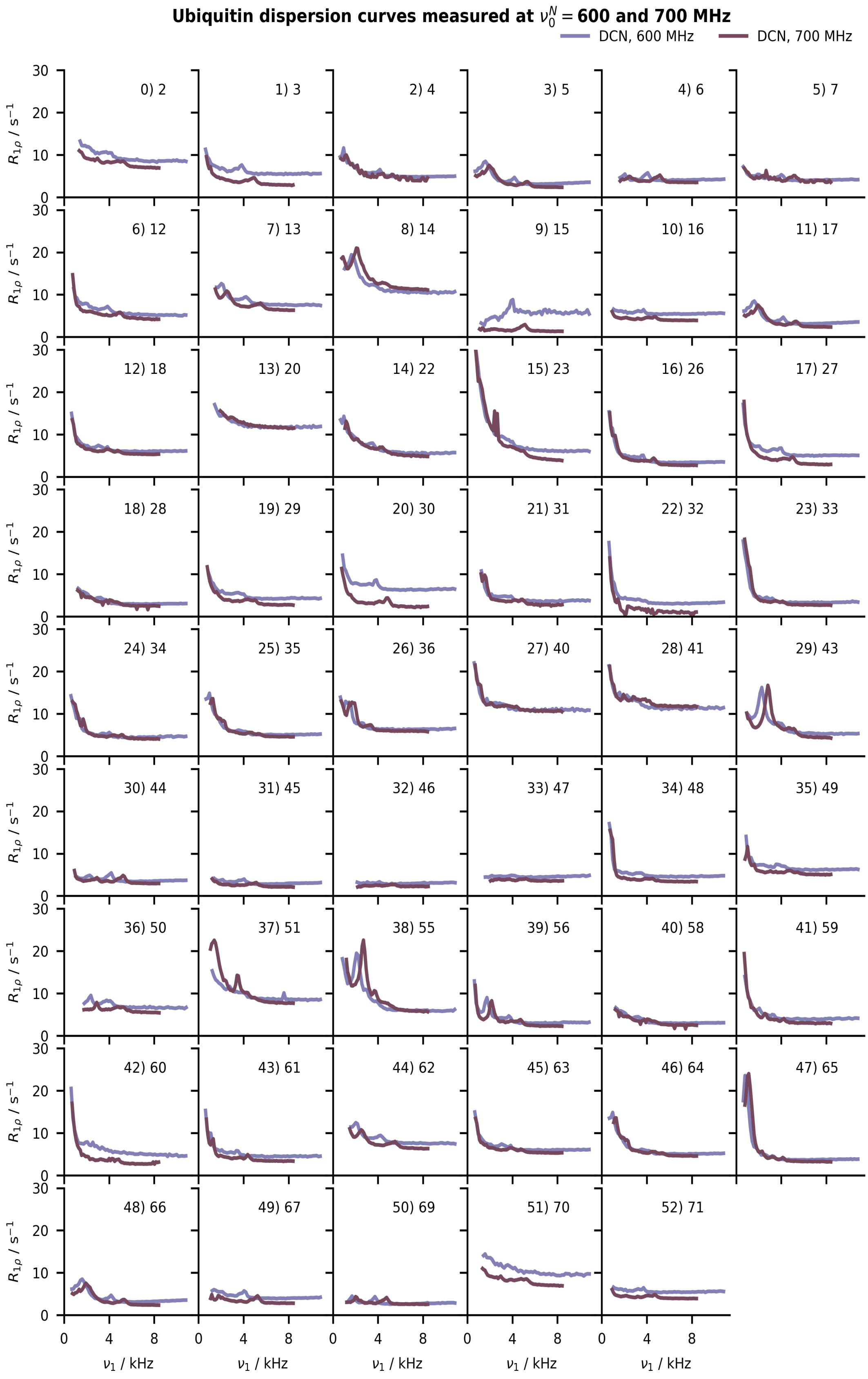


**Figure S9:** All dispersion curves measured for perdeuterated ubiquitin at 55.55 kHz MAS rate at $^1$H Larmor frequencies of 600 MHz and 700 MHz.

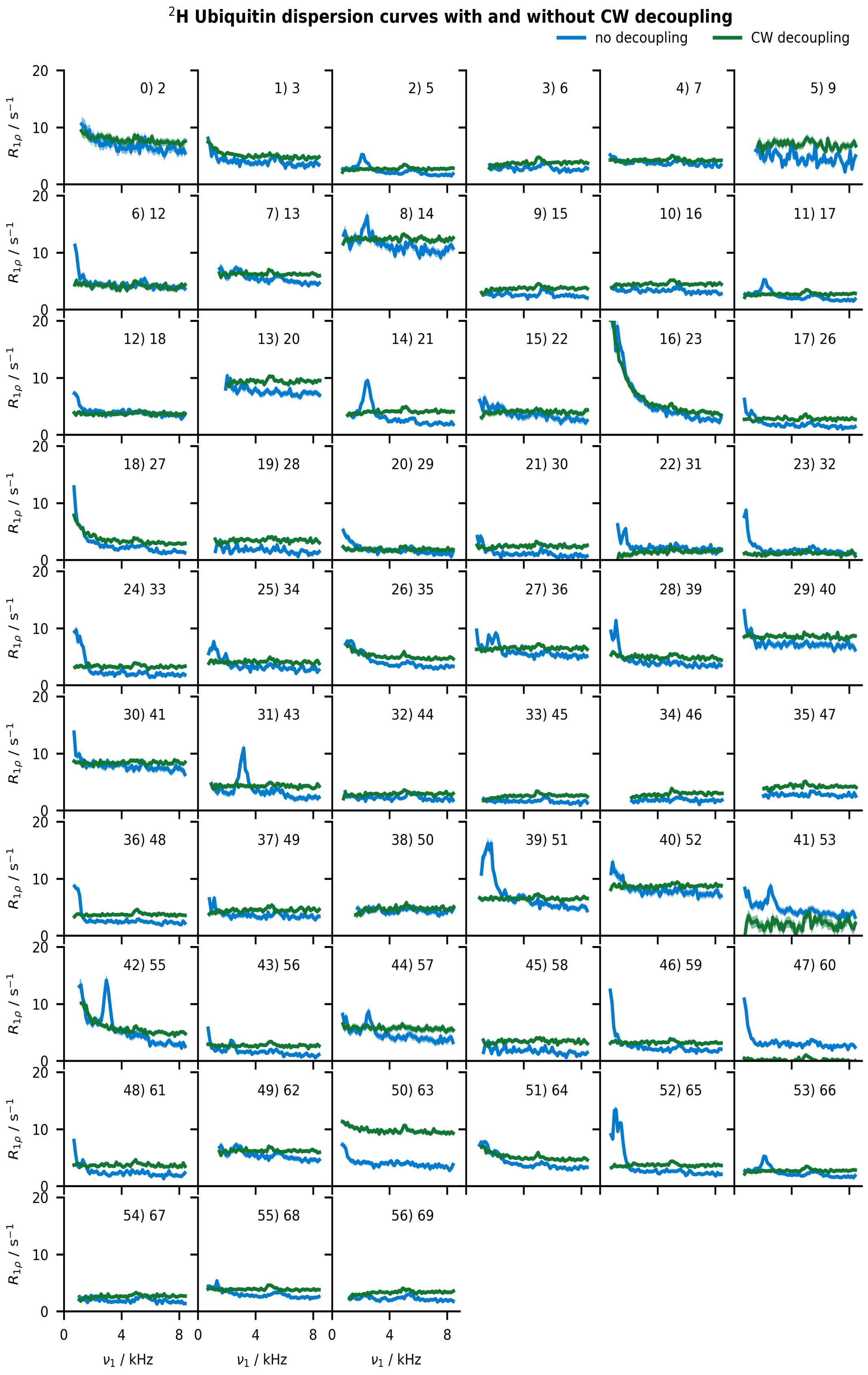


**Figure S10:** All dispersion curves measured in perdeuterated ubiquitin with and without CW decoupling at 100 kHz MAS and a $^{1}$H Larmor frequency of 700 MHz.

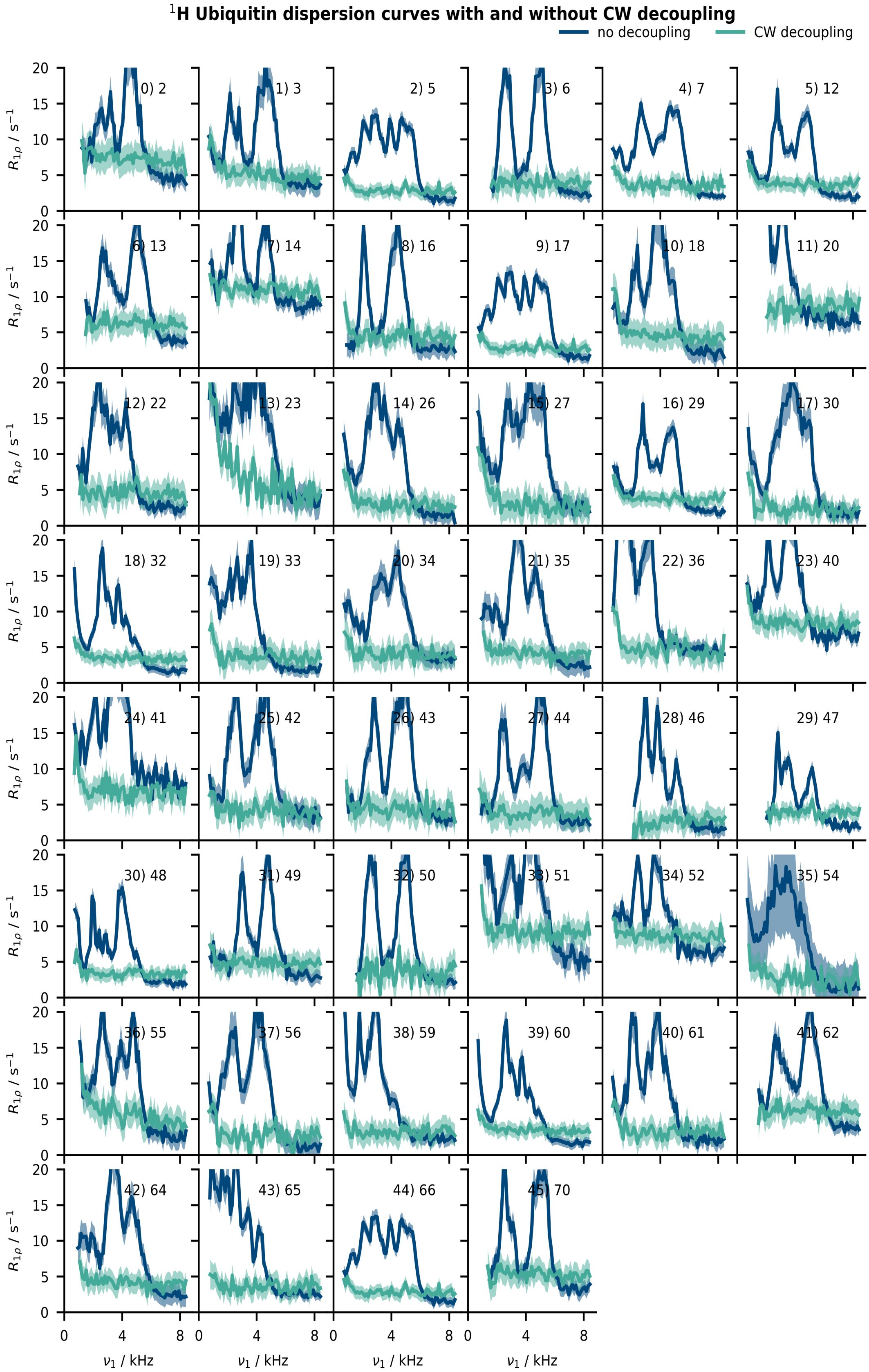


**Figure S11:** All dispersion curves measured in protonated ubiquitin with and without CW decoupling at 100 kHz MAS and a $^{1}$H Larmor frequency of 700 MHz.

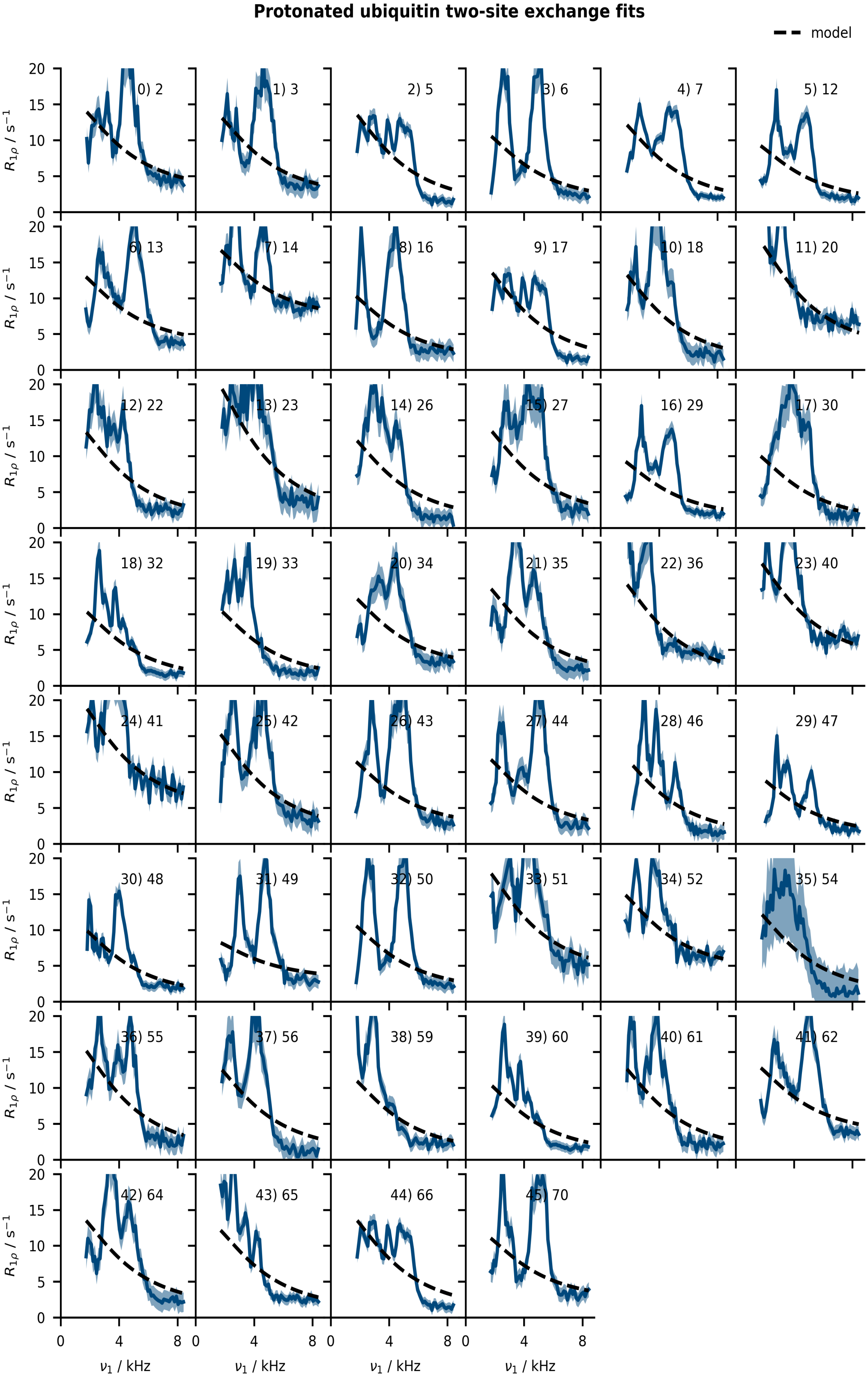


**Figure S12:** Two-site exchange fits to $^1$H ubiquitin data at 100 kHz MAS.

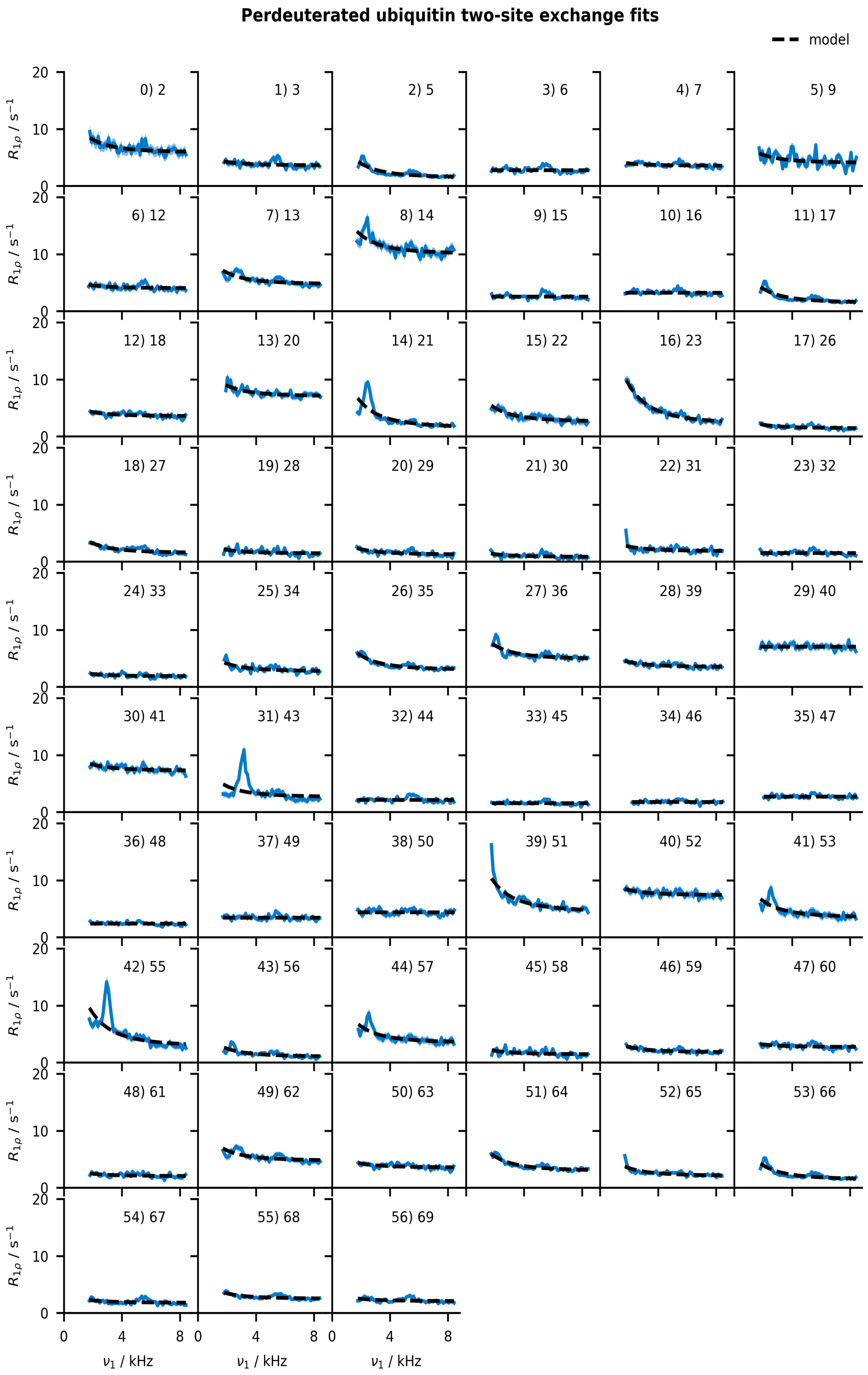


**Figure S13:** Two-site exchange fits to $^2$H ubiquitin data at 100 kHz MAS.

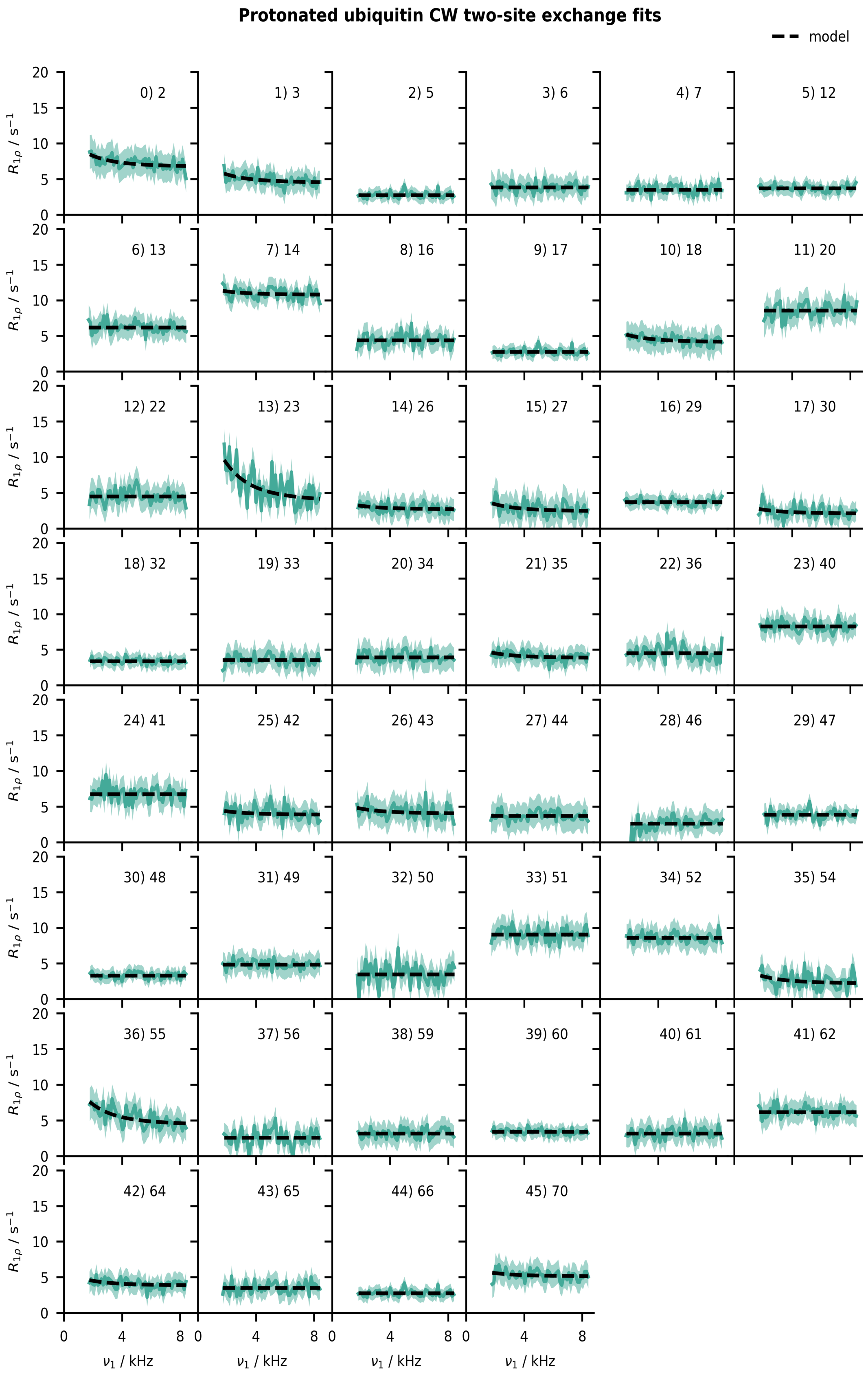


**Figure S14:** Two-site exchange fits to $^1$H ubiquitin data at 100 kHz MAS with 16 kHz CW decoupling.

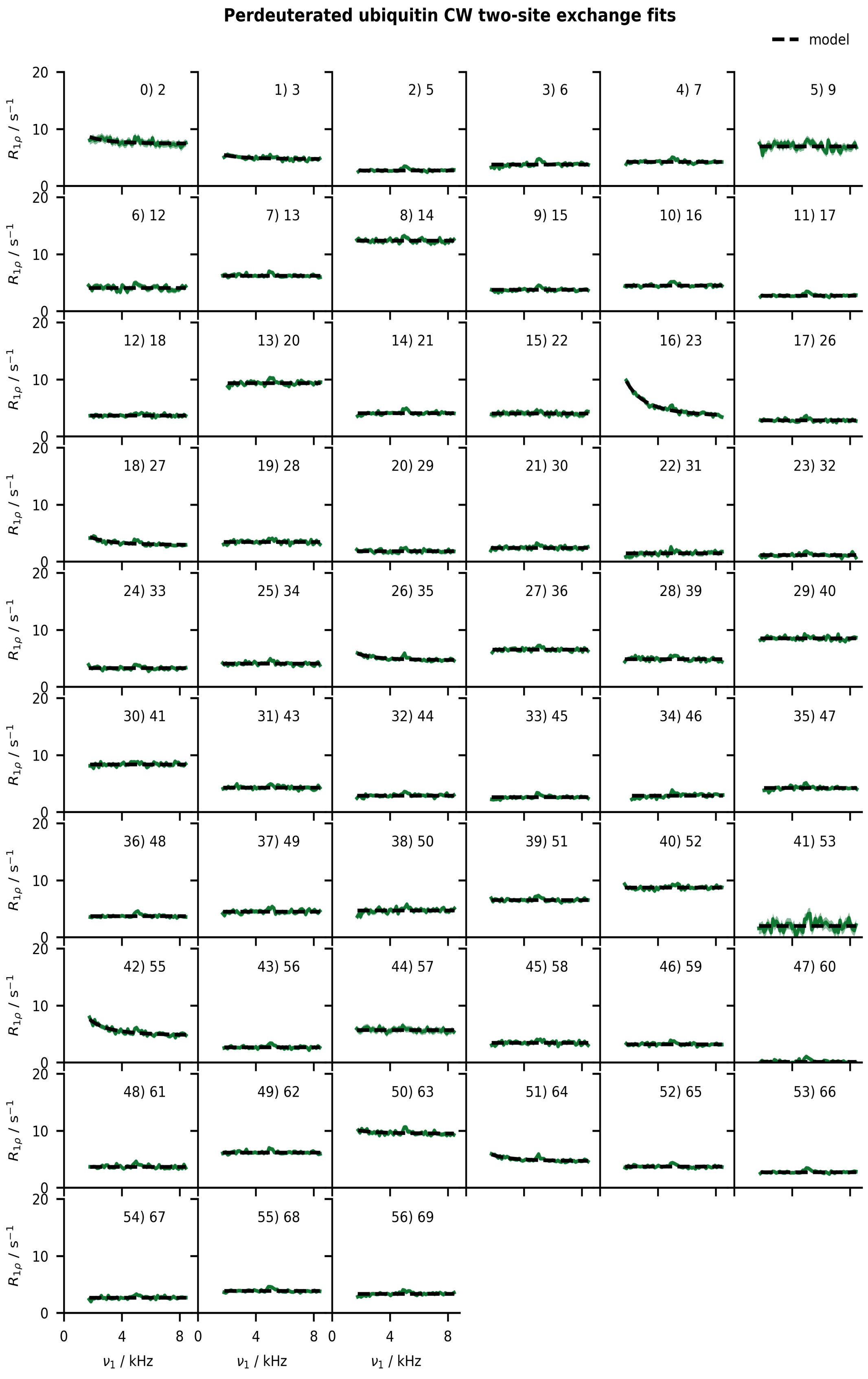


**Figure S15:** Two-site exchange fits to $^2$H ubiquitin data at 100 kHz MAS with 16 kHz CW decoupling.
